# Excretory/secretory products from *Hymenolepis nana* adult worms alleviate ulcerative colitis in mice via tuft/IL-13 signaling pathway

**DOI:** 10.1186/s13071-025-06893-x

**Published:** 2025-06-20

**Authors:** Rong Mou, Xuanyin Cui, Hongyan Wang, Zhenfen Zhang, Yi Cheng, Wenlan Wu, Jinfu Li, Ke Zhang

**Affiliations:** 1 The Guizhou Key Laboratory of Microbio and Infectious Disease Prevention & Control/The Key and Characteristic Laboratory of Modern Pathogenicity Biology, Department of Parasitology, School of Basic Medicine, Guizhou Medical University, Room 220, E-1 Building, Ankang Avenue No. 6, Guiyang, 561113 China; 2 Department of Histology and Embryology, School of Basic Medicine, Guizhou Medical University, Guiyang, 561113 China

**Keywords:** *Hymenolepis nana*, Ulcerative colitis, Excretory/secretory products, Tuft/IL-13, Intestinal stem cell

## Abstract

**Background:**

*Hymenolepis nana* (*H*. *nana*) is a zoonotic parasitic worm that parasitizes the small intestines of humans and rodents. Ulcerative colitis (UC) is a chronic and recurrent inflammatory bowel disease. Current symptom-based clinical treatments do not alter the natural course of UC, and mucosal healing has become a primary therapeutic goal for UC. However, the regulatory role of excretory/secretory products (ESPs) from *H*. *nana* adult worms in repairing the damaged intestinal mucosal barrier remains unclear.

**Methods:**

This study investigated the protective effects of ESPs on intestinal mucosal integrity by using a dextran sulfate sodium (DSS)-induced colitis mouse model and a mouse small intestine organoid inflammation model. Histopathological alterations of mouse intestinal tissues were determined by pathological staining; the alterations in mucins, tight junction proteins, cytokines, and the number of various intestinal cells were detected by Western blotting (WB), immunohistochemistry (IHC), immunofluorescence (IF) and real-time quantitative polymerase chain reaction (RT-qPCR), etc.

**Results:**

ESPs significantly improved DSS-induced intestinal damage in mice. Meanwhile, ESPs increased mucins and tight junction proteins expression and promoted intestinal stem cell proliferation and differentiation, thereby maintaining intestinal mucosal barrier integrity and alleviating UC in mice. In the DSS-induced inflamed small intestinal organoid model, ESPs reduced organoid damage and promoted the proliferation and differentiation of intestinal stem cells. The protective mechanism of ESPs might be related to the activation of the tuft/IL-13 signaling pathway, regulating intestinal barrier function and promoting the regeneration of intestinal stem cells.

**Conclusions:**

In conclusion, *H*. *nana*-derived ESPs intervention facilitates healing of intestinal mucosa to alleviate UC in mice, enriching the feasibility and selectivity of “helminthic therapy.”

**Graphical abstract:**

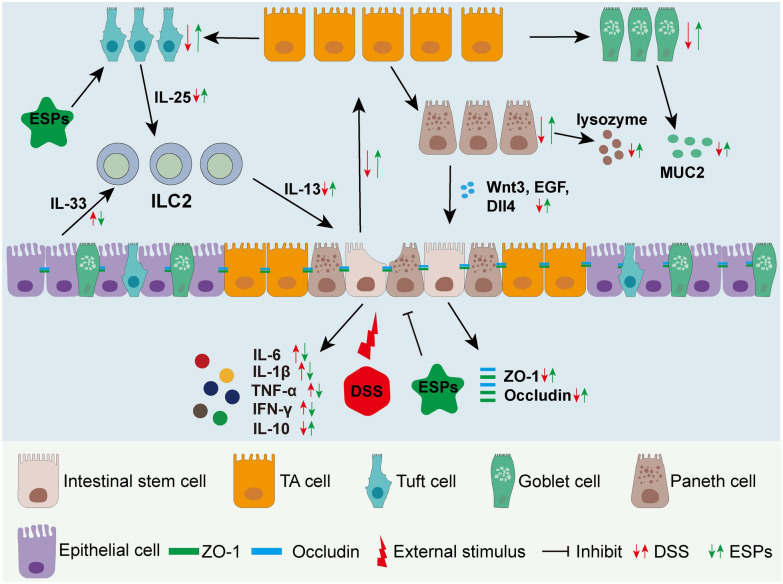

**Supplementary Information:**

The online version contains supplementary material available at 10.1186/s13071-025-06893-x.

## Background

Ulcerative colitis (UC) is an immune-mediated chronic inflammatory disease characterized by severe gastrointestinal symptoms, such as abdominal pain, diarrhea, and hematochezia. The disease has a prolonged course with recurrent episodes [1]. The global incidence of UC continues to rise, making it a significant health concern worldwide [2]. The pathogenesis of UC is closely related to persistent intestinal inflammation, which leads to mucosal necrosis and increased intestinal permeability, ultimately compromising the integrity of the intestinal mucosal barrier [3]. Mucosal healing has become the primary therapeutic goal for UC, and this is achieved through the regeneration and repair of the intestinal epithelium [4].

Intestinal epithelial cells undergo rapid renewal every 3–5 days [5]. The proliferation and differentiation of intestinal stem cells (ISCs) play a crucial role in the regeneration and repair of the intestinal epithelium [6]. ISCs are located at the base of the intestinal crypts and give rise to transit amplifying (TA) cells, which differentiate into all types of intestinal epithelial cells, including enterocytes, enteroendocrine cells, tuft cells, goblet cells, and Paneth cells [7]. The regulation of signaling pathways between tuft cells and type II innate lymphoid cells (ILC2) has garnered significant attention in parasitic intestinal infections. Tuft cells can recognize infections from *Nippostrongylus brasiliensis*, *Trichinella spiralis*, and *Helicotylenchus*, releasing IL-25 to increase ILC2 numbers and subsequently IL-13 secretion, which acts on ISCs to differentiate into more tuft and goblet cells [8–10]. Intestinal organoids mimic the crypt–villus structure and replicate various types of intestinal epithelial cells, serving as an in vitro model system for studying pathways and mechanisms of epithelial injury and repair [11, 12]. Over the past decade, intestinal organoid models derived from ISC have significantly advanced research on inflammatory bowel diseases [13, 14].

Currently, clinical medications for UC come with certain side effects and have relatively low remission rates [15]. Existing treatment options often fail to achieve satisfactory clinical efficacy. In recent years, there has been growing interest in leveraging the immunoregulatory capacity of helminths to develop new therapies for inflammatory and allergic diseases. Helminths are masters of immunoregulation, as they possess the ability to modulate the host’s immune system to support their own survival [16]. There is growing evidence that parasitic infections can regulate the host’s immune system, thereby reducing the incidence of UC [17]. Unlike many pathogens, parasitic helminths establish persistent infections that often elicit minimal inflammatory responses. Helminths mainly achieve immune modulation by releasing excretory/secretory products (ESPs) to evade immune-mediated pathology in the host [18].

*Hymenolepis nana* (*H*. *nana*) is a zoonotic parasitic helminth that infects the small intestine of humans and rodents, often causing mild inflammatory responses. Research on *H. nana* has primarily focused on its epidemiology, clinical manifestations, and treatment [19]. Previously, we found that *H*. *nana* infection and intraperitoneal injection of ESPs induced ISCs to differentiate into various intestinal epithelial cells, and these cells could secrete mucins, cytokines, and antimicrobial peptides to maintain intestinal homeostasis, which might have a palliative effect on UC [20]. Furthermore, it remains unclear how *H*. *nana* and its ESPs maintain intestinal mucosal integrity, particularly regarding their regulatory relationship with ISCs.

In this study, a dextran sulfate sodium (DSS)-induced colitis mouse model and a co-cultured system of mouse intestinal organoids and intestinal lamina propria lymphocytes (LPLs) were established to investigate the protective effects of ESPs on ISC regeneration and intestinal mucosal integrity. We investigated whether ESPs could alleviate UC in mice by promoting ISC proliferation and differentiation through the tuft/IL-13 signaling pathway and protecting the intestinal mucosal barrier integrity.

## Methods

### Main reagents

Reagents sources can be found in Reagents in the Supplementary Materials (Additional file 1).

Primary antibodies for ZO-1 (21773–1-AP), β-tubulin (10094–1-AP), and occludin (66378–1-IG) were offered by Proteintech (Wuhan, China); those for Lgr5 (R380973) were obtained from ZenBio (Chengdu, China); those for IL-13 (A00077-2) were offered by Boster (Wuhan, China); those for GAPDH (AF7021) were purchased from Affinity (Jiangsu, China); those for MUC2 (ab272692), lysozyme (Lyz) (ab108508), MMP7 (ab302893), and Dclk1 (ab31704) were obtained from Abcam (Cambridge, UK); those for Lgr5 (DF2816) were purchased from Affinity (Jiangsu, China); and those for PCNA (PA5-27214, MA5-11358) and fluorescent secondary antibody anti-rabbit 488 (A21206) were offered by Thermo Fisher Scientific (Waltham, MA, USA). Secondary antibodies (HY-P8001 and HY-P8004) were purchased from MCE (Shanghai, China).

### Animal experiments

#### Acquisition of *H*. *nana* from hamsters and the extraction and identification of excretory/secretory products (ESPs) from *H*. *nana* adult worms

Hamsters (*n* = 40) that were 4–6 weeks old were purchased from the pet market in Nanming District, Guiyang, China, in May 2024 (Additional file 2: Fig. S1A). Hamsters were anesthetized and executed, and the small intestine was dissected to obtain intestinal parasites (all hamsters were infected with parasites).

The ESPs were extracted using the method previously reported by our group [20] (specific steps are included in Additional file 1). The methodology for the identification of *H*. *nana* and ESPs is presented in Additional file 1, and the results are presented in Additional file 2: Fig. S1B–G.

#### Experiments in C57BL/6 J mice

Healthy adult male C57BL/6 J mice (6–8 weeks old; 22 ± 2 g) were obtained from the Experimental Animal Center of Guizhou Medical University (SCXK (Jing) 2019–0010). Animals were kept under specific pathogen-free conditions at room temperature of 20–22 °C, and subjected to a controlled 12-h light/dark cycle. All mice were given adaptive feeding for at least 1 week before formal experimentation.

The UC animal model and the intervention approach were based on previous studies [21, 22]. Mice were randomly assigned to four groups (*n* = 10/group): control (Ctrl), ESPs, DSS, and DSS + ESPs groups. The DSS and DSS + ESPs groups were provided with sterilized 4% (m/v) DSS dissolved in H_2_O for 7 consecutive days. Pre-experimentation found that 50 μg/day of ESPs was the most effective (Additional file 3: Fig. S2), so this dose was chosen for subsequent experiments. The ESPs and DSS + ESPs groups were injected intraperitoneally with 50 μg/day of ESPs seven times per mouse. On the eighth day, all mice were anesthetized and euthanized.

#### Disease activity index (DAI) score

During the experiment, body weight, shape of feces, and fecal occult blood were monitored every day to score the DAI (specific steps of the fecal occult blood test are described in Additional file 1). The DAI was calculated using the following formula:

DAI = (weight change score + loose stools score + bloody stools score)/3 [23].

The scores were yielded according to Additional file 4: Table S1.

#### Mouse small intestine organoid experiments

Crypt and intestinal lamina propria lymphocytes (LPLs) were extracted according to the previous studies [24, 25] (specific steps are listed in Additional file 1). After mixing extracted crypts with Matrigel (the percentage of Matrigel should be greater than 70%), a 30 μL drop was placed in the center of each well of a 24-well plate. The plate was then incubated at 37 °C with 5% CO_2_ for 20 min to allow the Matrigel to solidify. After gel solidification, 500 μL of complete small intestinal organoid culture medium was added to each well. Organoids were successfully cultured within 5–7 days. The organoid inflammation model was established according to the literature [26]. The isolated LPLs were cultured with the passaged organoids at a ratio of 7:1 in Matrigel. After 3 days of culturing, DSS 4 μM was added and incubated for 24 h, and then co-cultured with different doses of ESPs for an additional 24 h. The morphology of the organoids was observed under an inverted microscope (DS-Ri2, Nikon Ltd, Japan). Mouse small intestinal organoids were collected and stored at −80 °C for subsequent experiments (specific steps are presented in Additional file 1).

### Hematoxylin–eosin (H&E) staining, alcian blue–periodic acid–Schiff (AB-PAS) staining, and Masson’s trichrome (Masson) staining

Pathological staining of mouse intestinal tissues was performed according to the method reported by our group [20, 27] (specific steps are described in Additional file 1). H&E staining was used for histopathologic examination of inflammatory conditions of the colon and ileum, AB-PAS staining for detection of goblet cells in the colon and ileum, and Masson staining for collagen fibers in the colon.

### Myeloperoxidase (MPO) activity assay

MPO activity was determined according to the myeloperoxidase assay kit. Briefly, the mouse colon tissues were ground to 5% tissue homogenate, and we then proceeded according to the instructions of the kit, and the absorbance value was measured at 460 nm on an enzyme meter. MPO activity was expressed as U/g in the tissue [28].

### Real-time quantitative polymerase chain reaction (RT-qPCR)

For mouse intestinal tissues and intestinal organoids, the total RNA was extracted and then reversely transcribed to cDNA following the method reported by our group [27] (specific steps are presented in Additional file 1). The relative mRNA transcription levels of *Lgr5*, *Dclk1*, *MUC2*, *Wnt3*, *EGF*, *Dll4*, *Lyz1*, *IL-13*, *IL-25*, *IL-33*, *IL-6*, *IL-1β*, *TNF-α*, *IFN-γ*, and *IL-10* were calculated by the 2^-^^ΔΔCt^ method, and *GAPDH* was used as the internal control for normalization. The primer sequences are listed in Additional file 5: Table S2.

### Immunohistochemistry (IHC) and immunofluorescence (IF)

IHC and IF staining of mouse intestinal tissues were performed according to the specific references [27, 29] (specific steps are listed in Additional file 1). For IHC, the sections were incubated overnight with primary antibodies specific for ZO-1 (1:2,000), occludin (1:200), MUC2 (1:2,000), Olfm4 (1:200), Dclk1 (1:200), PCNA (1:200), MMP7 (1:200), lysozyme (Lyz) (1:2,000), and IL-13 (1:200). For IF, the sections were incubated overnight with primary antibodies respectively specific for Lgr5 (1:100), PCNA (1:200), Olfm4 (1:200), MUC2 (1:500), Dclk1 (1:200), MMP7 (1:400), and lysozyme (Lyz) (1:250).

### Western blotting (WB)

The mouse colonic, ileum, and small intestinal organoid tissues were digested with radioimmunoprecipitation assay (RIPA) lysis buffer. After measuring the concentration, an equal amount of protein samples was loaded and isolated using 8–12% sodium dodecyl sulfate polyacrylamide gel electrophoresis (SDS-PAGE). Next, the proteins were transferred onto a polyvinylidene difluoride (PVDF) membrane. After blocking with 5% non-fat milk, the membrane was separately incubated with the primary antibodies ZO-1 (1:20,000), occludin (1:10,000), Lgr5 (1:1,000), Dclk1 (1:1,000), Lyz (1:11,000), MUC2 (1:1,000), IL-13 (1:1,000), and PCNA (1:2,000) at 4 °C overnight and then incubated with the secondary antibody (1:10,000) for 1 h at room temperature. Finally, Super ECL Detection Reagent was utilized for sensitive detection of the protein and observed using a chemiluminescence imager (Bio-Rad, Hercules, CA, USA).

### Statistical analysis

The statistical analysis and graphical representation were performed using GraphPad Prism (Version 5.0). The data were presented as mean + standard deviation (SD). Semi-quantitative statistics used Image J software (version 1.53i, US National Institutes of Health, USA) after image acquisition. Measurements were first subjected to normality tests, and the homogeneity of variance test was performed between groups. Mann-Whitney *U-*test was employed as a non-parametric statistical method to analyze data that deviated from normal distribution. One-way analysis of variance (ANOVA) was used to test differences between multiple groups, and the Mann–Whitney *U*-test and *t*-test were used to compare differences between two groups. A *p*-value of less than 0.05 meant a statistically significant difference.

## Results

### ESPs intervention attenuates the clinical signs induced by DSS in mice

**Fig. 1:**
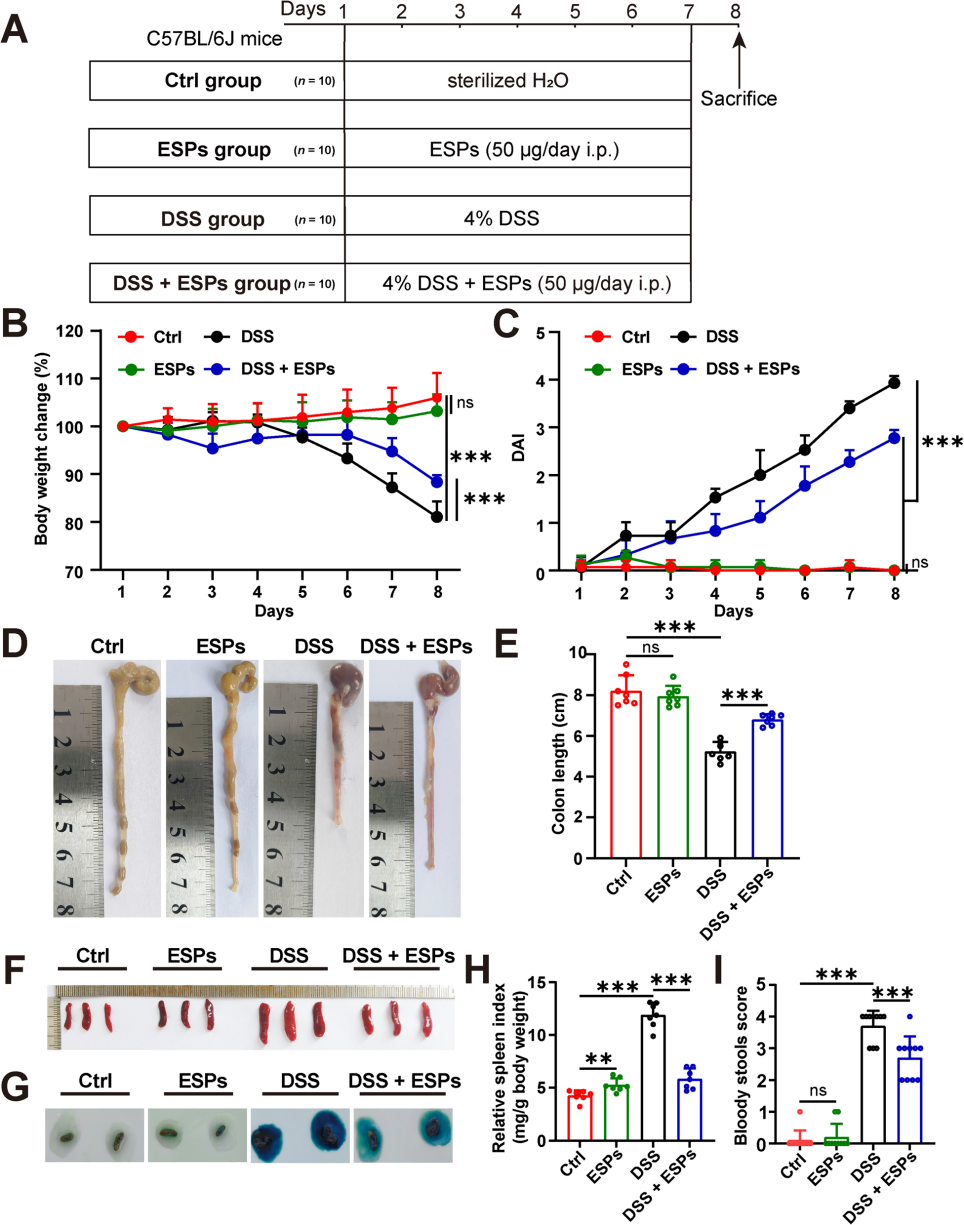
ESPs improve the clinical symptoms of DSS-induced colitis in mice. **A** Experimental flowchart. The mice of DSS and DSS + ESPs groups were given 4% DSS in sterilized H_2_O (ad libitum) for 7 days; the mice of the ESPs and DSS + ESPs groups were intraperitoneally (i.p.) injected with 50 μg/day of ESPs for 7 days; the mice in the Ctrl group were given sterilized H_2_O (ad libitum) for 7 days. All mice were anesthetized and euthanized at the eighth day. **B** Changes in mouse body weight (%). Initial body weight was set as 100%. **C** Disease activity index (DAI) score. **D** Representative colon images of four groups. **E** Colon length (cm). **F** Representative spleen images of four groups. **G** Representative pictures of fecal occult blood test results at the eighth day. **H** Statistics for spleen index. (**I**) Statistics for the bloody stools score at the eighth day. Data are presented as mean + SD in (**B**), (**C**), (**E**), (**H**), and (**I**). Group size of *n* = 10 per group for (**B**), (**C**), and (**I**) and *n* = 7 per group for (**E**) and (**H**). ^**^p < 0.01; ^***^p < 0.001; ns, not statistically significant

To assess whether ESPs provided protective effects against DSS-induced colitis, we established a DSS-induced colitis mouse model and administered ESPs via intraperitoneal injection (Fig. 1A). On the basis of body weight change, colon length, spleen index, and pathological alterations, we found that ESPs alleviated UC in a dose-dependent manner in mice, with the best effect at 50 μg/day. Therefore, this dose was selected for subsequent experiments (Additional file 3: Fig. S2). By evaluating the changes of weight loss, colon shortening, and intestinal bleeding, we monitored the progression of UC in mice. We observed that DSS caused weight loss, colon shortening, spleen enlargement, and intestinal bleeding in the mice, while ESPs intervention alleviated these changes (Fig. 1B–I). These findings indicate that ESPs significantly improve the clinical symptoms of UC in mice.

### ESPs alleviate the pathological damage to the colon in UC mice

**Fig. 2:**
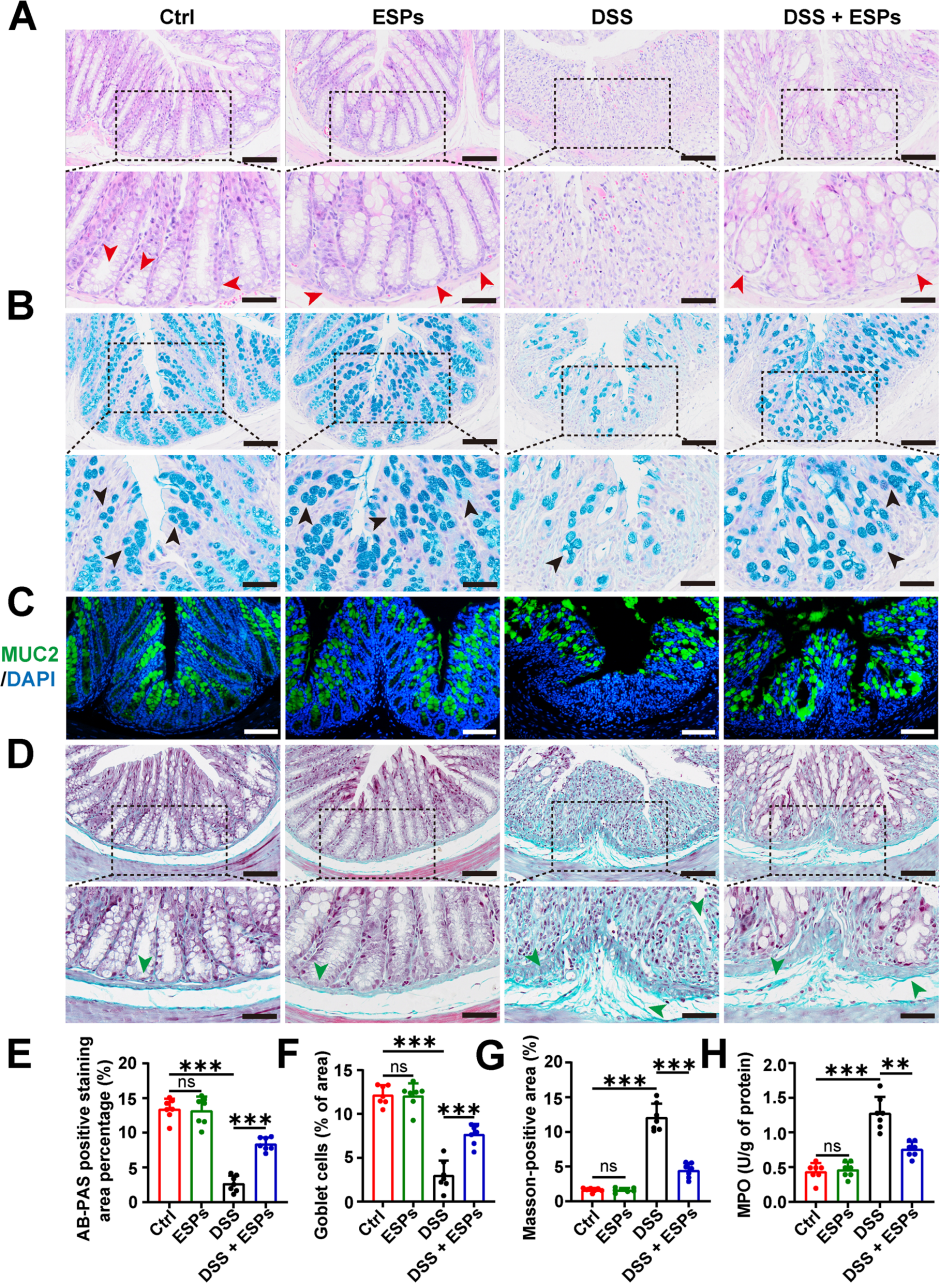
ESPs attenuate DSS-induced pathological damage in the mouse colon intestine. **A** Representative images of H&E-stained colon intestine (the crypts indicated by red arrowheads in the lower panel; scale bars 100 μm for the upper panel and 50 μm for the lower panel). **B** Representative images of AB-PAS-stained goblet cells (deep blue indicated by black arrowheads in the lower panel; scale bars 100 μm for the upper panel and 50 μm for the lower panel). **C** Representative images of IF for MUC2 (green) and the nucleus (4′,6-diamidino-2-phenylindole (DAPI), blue) (scale bars 100 μm). **D** Representative images of Masson staining (the collagen fibers indicated by green arrowheads in the lower panel; scale bars 100 μm for the upper panel and 50 μm for the lower panel). Percentages of the statistics of AB-PAS-stained positive area (**E**), the number of goblet cells (**F**), and the Masson-positive area (**G**) were semi-quantified using Image J. **H** Colon MPO activity (U/g) was measured by myeloperoxidase assay kit. Data are presented as mean + SD for **E**–**H**. Group size of *n* = 7 per group. ^**^p < 0.01; ^***^p < 0.001; ns, not statistically significant

H&E staining was used to observe mucosal ulcers and crypt integrity in colon tissues, AB-PAS staining and IF were used to assess the number of goblet cells, and Masson staining was used to evaluate collagen fiber alterations. MPO activity in colonic tissues is an important index reflecting the accumulation of neutrophils in the tissues, and it was measured to determine inflammation. The H&E staining results showed that the ESPs group exhibited no significant pathological changes, while the DSS group exhibited marked colon tissue damage, including mucosal ulcers and impaired crypt integrity. After ESPs intervention, colon tissue damage in the mice was significantly improved (Fig. 2A). Compared with the Ctrl group, the ESPs group showed no significant changes in goblet cell numbers, collagen fibers, and MPO activity. In contrast, the DSS group showed a dramatic decrease in goblet cell numbers, increased fibrosis as shown by Masson staining, and elevated MPO activity. In the DSS + ESPs group, goblet cell numbers were partially restored, and both fibrosis and MPO activity were reduced (Fig. 2B–H). These results indicate that ESPs intervention can alleviate the intestinal histological damage to the colon induced by DSS in mice.

### ESPs improve the intestinal barrier function of the colon in UC mice

**Fig. 3:**
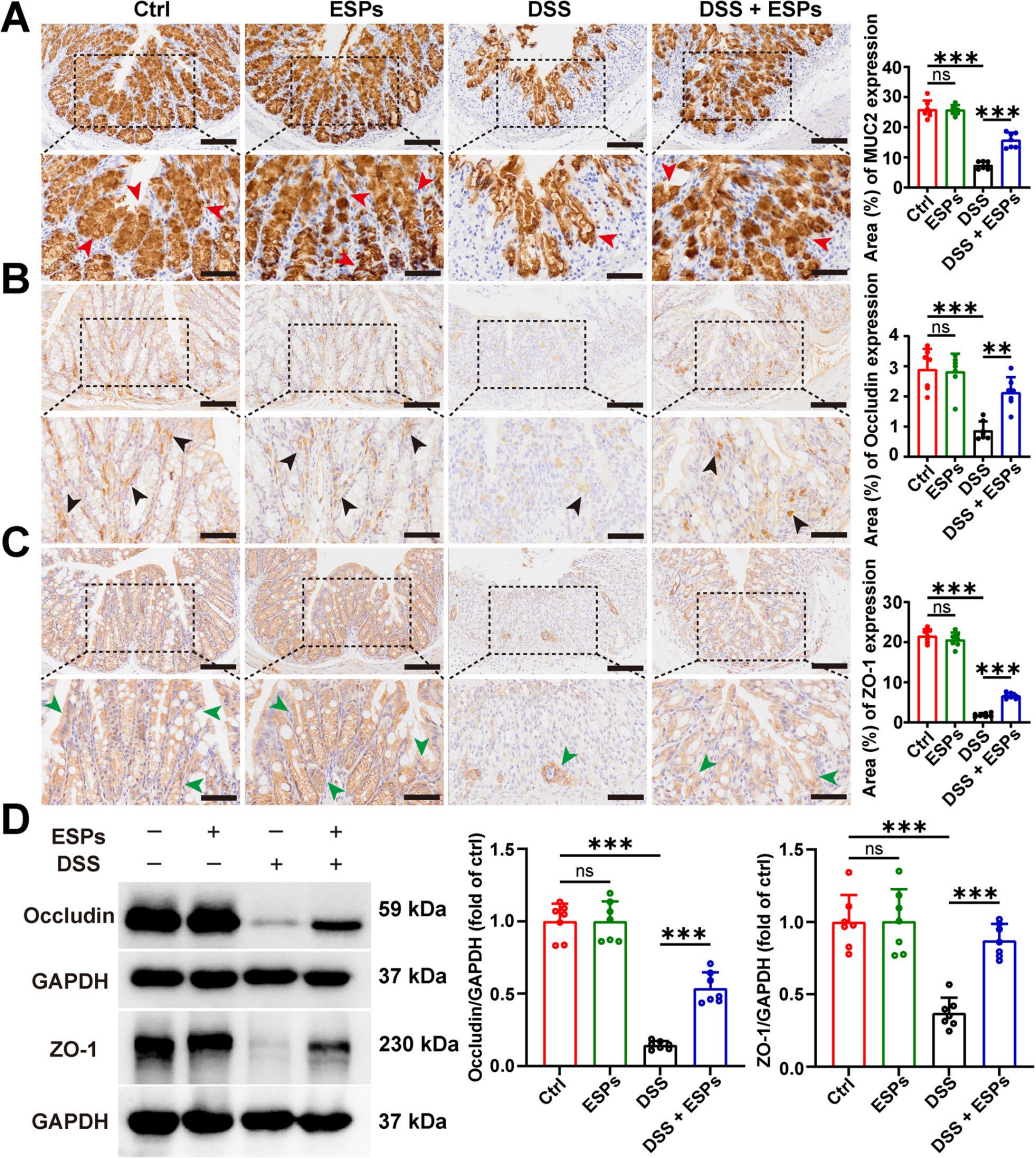
ESPs improve intestinal barrier integrity in the colon of UC mice. Representative images of IHC for MUC2 (**A**), occludin (**B**), and ZO-1 (**C**); percentages of MUC2, occludin, and ZO-1-positive areas were semi-quantified using Image J; positive MUC2, occludin, and ZO-1 were stained brown and indicated by red, black, and green arrowheads in the lower panels, respectively (scale bars 100 μm for the upper panel and 50 μm for the lower panel). (**D**) WB results for occludin and ZO-1 were set as the left panel and relative expressions of occludin and ZO-1 were semi-quantified using Image J as the right panel. Data are presented as mean + SD for the right panel of (**A**–**D**). Group size of *n* = 7 per group. ^**^p < 0.01; ^***^p < 0.001; ns, not statistically significant

To explore the impact of ESPs on the intestinal barrier in UC mice, we examined the changes in mucin (MUC2) and tight junction proteins (occludin and ZO-1) by using IHC and WB. The results showed that, in the ESPs group, there were no significant changes in mucin and tight junction protein levels. In contrast, the DSS group exhibited a significant reduction in mucin, and as previously observed, the number of goblet cells was drastically decreased in the DSS group. This might be due to the excessive loss of goblet cells, leading to decreased mucin secretion (Fig. 3A). The DSS group also showed a marked reduction in tight junction proteins, while the DSS + ESPs group exhibited an increase in tight junction proteins expression (Fig. 3B–D). These results suggest that ESPs can improve DSS-induced intestinal barrier dysfunction by protecting mucin and tight junction proteins, thereby maintaining the integrity of the intestinal epithelium structure.

### ESPs promote the proliferation of ISC in the colon of UC mice

**Fig. 4:**
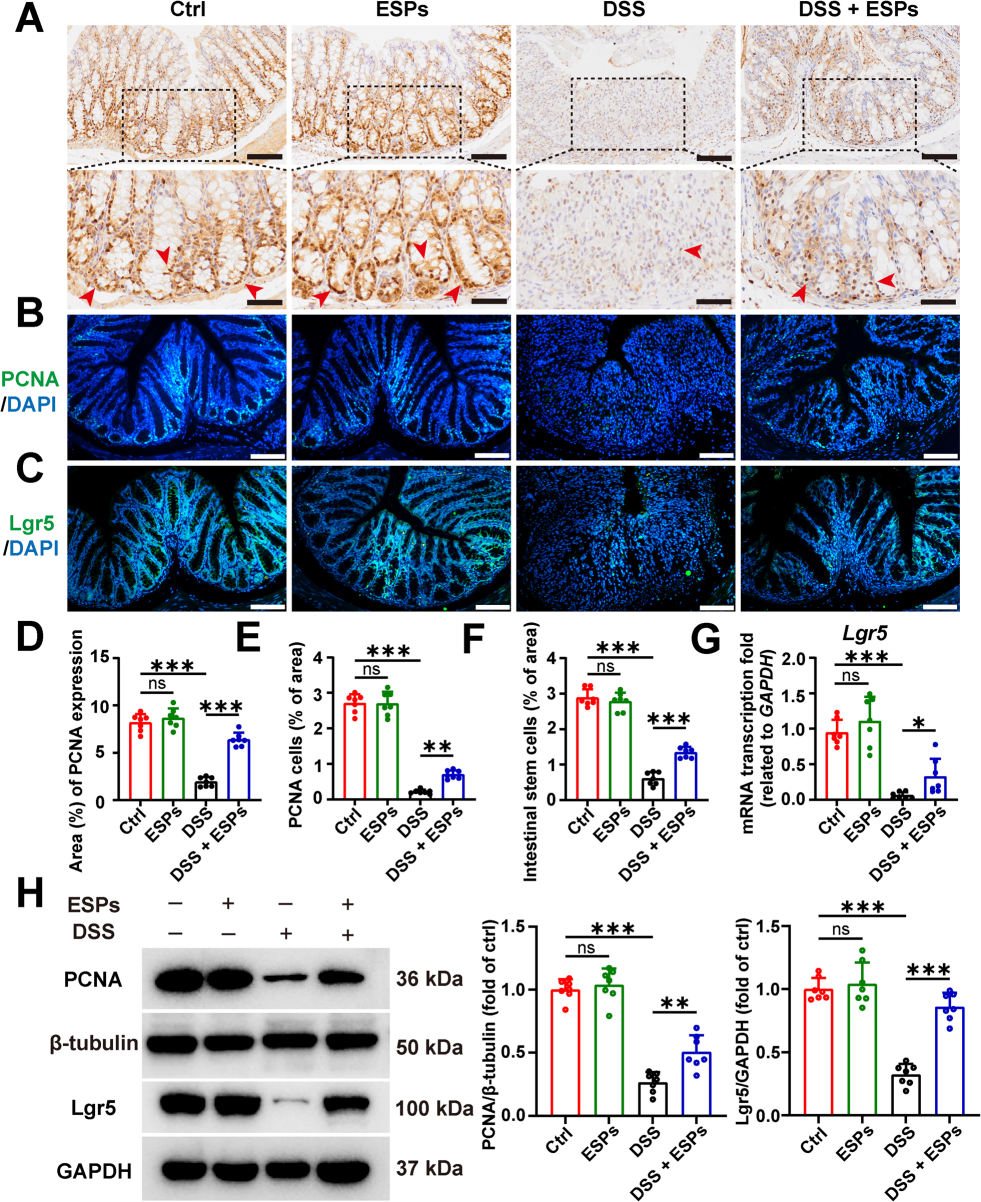
ESPs promote ISC proliferation in the mouse colon to alleviate UC. **A** Representative images of IHC for PCNA (brown indicated by red arrowheads in the lower panel, scale bars 100 μm for the upper panel and 50 μm for the lower panel). Representative images of IF for PCNA (**B**) (green) and Lgr5 (**C**) (green) and the nucleus (4′,6-diamidino-2-phenylindole (DAPI), blue) (scale bars 100 μm). Percentages of PCNA-positive area (**D**), the number of PCNA cells (**E**), and ISC (**F**) were semi-quantified using Image J. **G** The transcription level of *Lgr5* was determined using the 2^−ΔΔCt^ method normalized to *GAPDH*. **H** WB results for PCNA and Lgr5 were set as left panel and relative expressions of PCNA and Lgr5 were semi-quantified using Image J as right panel. Data are presented as mean + SD for **D**–**G** and the right panel of **H**. Group size of *n* = 7 per group. * *p* < 0.05; ** *p* < 0.01; *** *p* < 0.001; ns, not statistically significant

Intestinal epithelial cells are derived from the proliferation and differentiation of ISC. Using IHC, IF, and WB, we assessed the proliferating cell marker PCNA; IF, RT-qPCR, and WB were used to detect the ISC marker Lgr5. The results showed no change in PCNA-positive cells and ISCs in the ESPs group compared with the Ctrl group. In the DSS group, the number of cells was reduced, but after ESPs intervention, both PCNA-positive cells and ISCs were partially restored (Fig. 4). These results suggest that ESPs can promote the proliferation of colonic ISC and thus support intestinal epithelial cell regeneration.

### ESPs reduce lysozyme levels and inflammation responses in the colon of UC mice

**Fig. 5:**
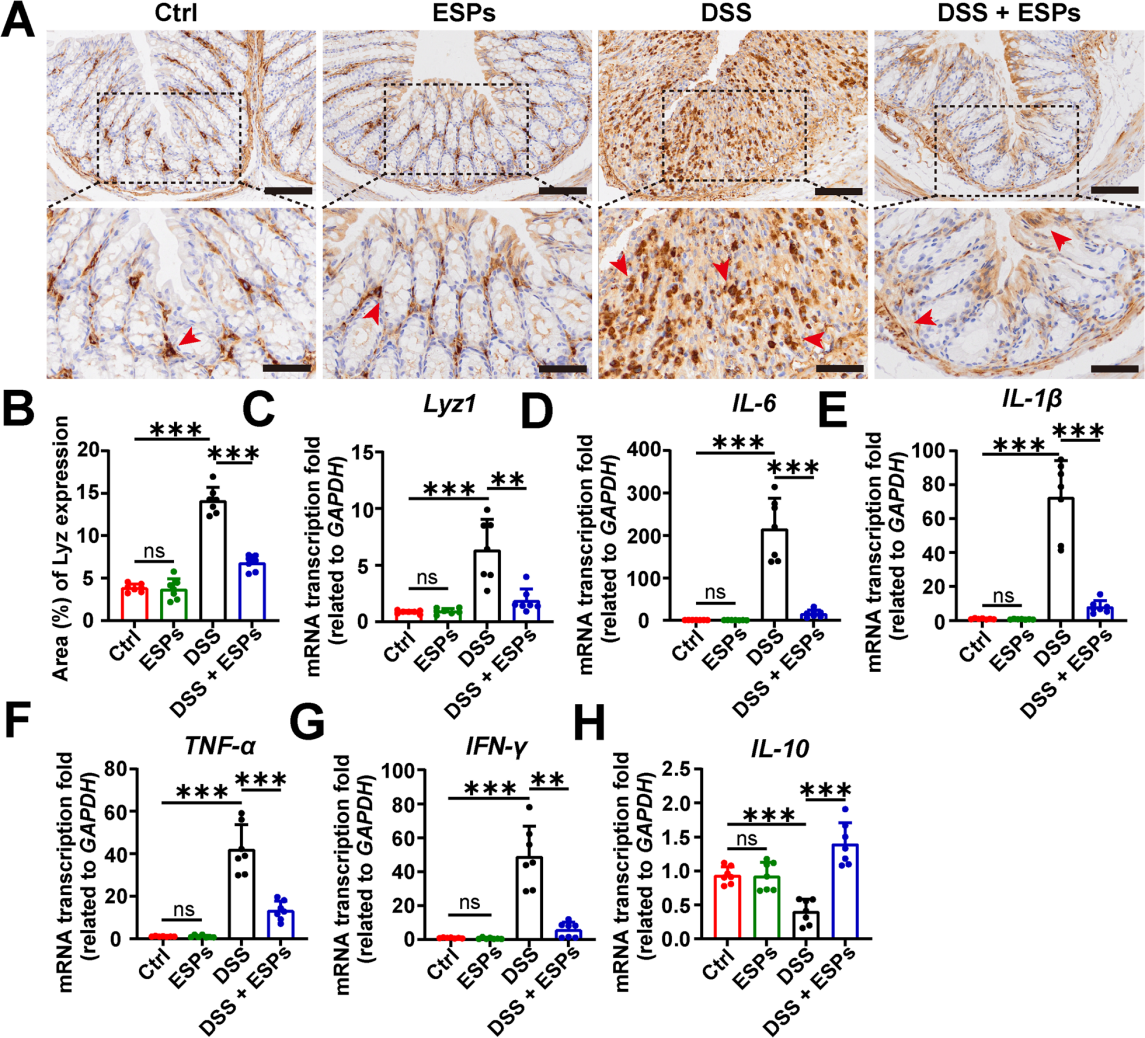
ESPs protect UC mice by inhibiting colonic lysozyme (Lyz) increase and inflammatory responses. **A** Representative images of IHC for Lyz (brown indicated by red arrowheads in the lower panel; scale bars 100 μm for the upper panel and 50 μm for the lower panel). (**B**) The percentage of Lyz-positive area was semi-quantified using Image J. The transcription levels of *Lyz1* (**C**), *IL-6* (**D**), *IL-1β* (**E**), *TNF-α* (**F**), *IFN-γ* (**G**), and *IL-10* (**H**) were determined using the 2^−ΔΔCt^ method normalized to *GAPDH*. Data are presented as mean + SD for **B**–**H**. Group size of *n* = 7 per group. ^**^p < 0.01; ^***^p < 0.001; ns, not statistically significant

The changes in colonic lysozyme levels were assessed by IHC and RT-qPCR. The results showed that the level of lysozyme did not change significantly in the ESPs group, but it increased in the DSS group and decreased in the DSS + ESPs group (Fig. 5A–C). Compared with the Ctrl group, the transcription levels of pro-inflammatory factors (*IL-6*, *IL-1β*, *TNF-α*, and *IFN-γ*) and anti-inflammatory factor *IL-10* in the ESPs group showed no significant changes. In the DSS group, these factors were significantly elevated, and the anti-inflammatory factor *IL-10* was decreased. ESPs intervention reduced the transcription levels of pro-inflammatory factors and upregulated the expression of anti-inflammatory factor (Fig. 5D–H). These results suggest that ESPs alleviate colon damage and intestinal inflammation by reducing lysozyme levels and modulating the balance between pro-inflammatory and anti-inflammatory factors.

### ESPs alleviate the pathological damage to the small intestine in UC mice

**Fig. 6:**
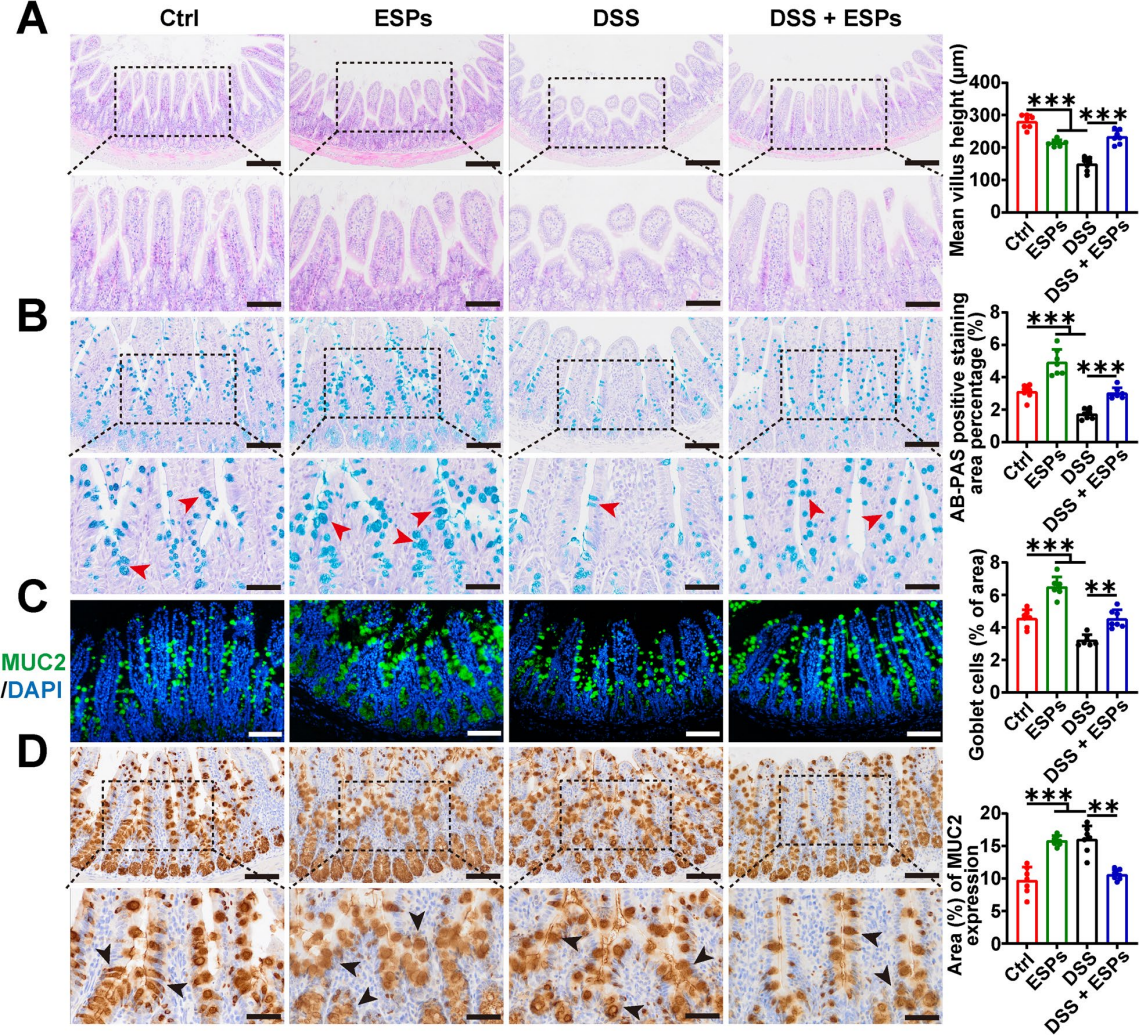
ESPs attenuate DSS-induced pathological damage in the mouse small intestine. **A** Representative images of H&E-stained small intestine; mean villus height (μm) was measured by Image J (scale bars 200 μm for the upper panel and 100 μm for the lower panel). **B** Representative images of AB-PAS-stained goblet cells; the statistic of AB-PAS-stained positive area was semi-quantified using Image J (deep blue indicated by red arrowheads in the lower panel; scale bars 100 μm for the upper panel and 50 μm for the lower panel). **C** Representative images of IF for MUC2 (green) and the nucleus (4′,6-diamidino-2-phenylindole (DAPI), blue) (scale bars 100 μm); the number of goblet cells was semi-quantified using Image J. **D** Representative images of IHC for MUC2; MUC2-positive area was semi-quantified using Image J (brown indicated by black arrowheads in the lower panel; scale bars 100 μm for the upper panel and 50 μm for the lower panel). Data are presented as mean + SD for the right panel of **A**–**D**. Group size of *n* = 7 per group. ^**^p < 0.01; ^***^p < 0.001

H&E staining was used to assess the damage to the ileum in UC mice. The results showed that both the ESPs and DSS groups had reduced villus height, but ESPs intervention alleviated the shortening of villus height caused by DSS (Fig. 6A). The mucus layer covers the gastrointestinal tract and provides barrier protection, serving as one of the first lines of immune defense. AB-PAS staining and IF were used to examine changes in goblet cell numbers, and IHC was used to detect changes in mucin MUC2. Compared with the Ctrl group, the ESPs group showed an increase in goblet cells and mucin. In the DSS group, the number of goblet cells was reduced, while mucin increased. ESPs intervention was able to increase the goblet cell numbers and suppress the excessive secretion of mucin caused by DSS (Fig. 6B–D). These results indicate that the mice can protect themselves by secreting mucin, and ESPs can alleviate the pathological damage to the ileum in UC mice induced by DSS.

### ESPs protect against DSS-induced reduction of tuft cells and IL-13 in mouse small intestine

**Fig. 7:**
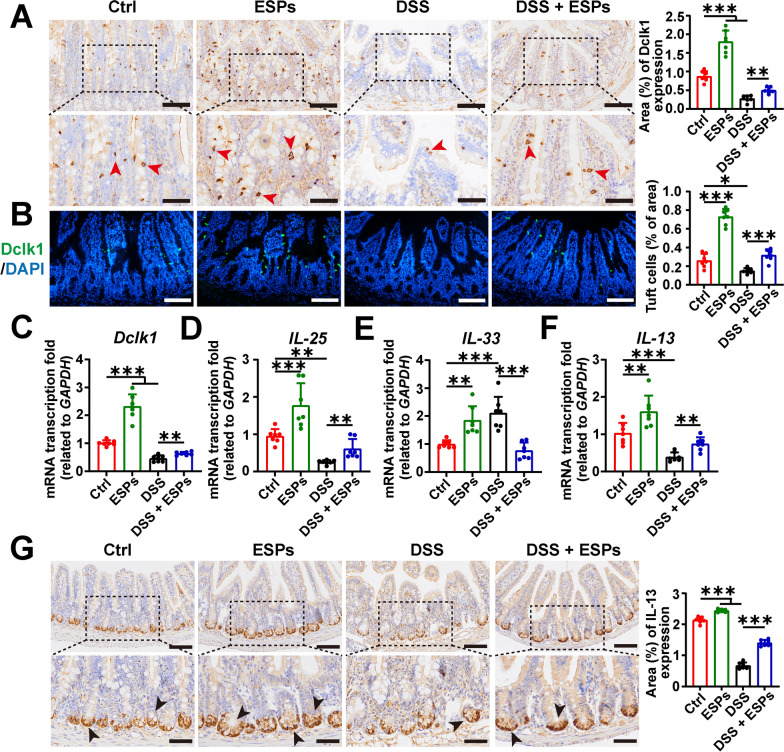
ESPs increase the number of tuft cells and IL-13 in the small intestine of UC mice. **A** Representative images of IHC for Dclk1 (brown indicated by red arrowheads in the lower panel; scale bars 100 μm for the upper panel and 50 μm for the lower panel) and (**B**) IF for Dclk1 (green) and the nucleus (4′,6-diamidino-2-phenylindole (DAPI), blue) (scale bars 100 μm). Percentages of Dclk1-positive area and the number of tuft cells were semi-quantified using Image J in **A** and **B**. The transcription levels of *Dclk1* (**C**), *IL-25* (**D**), *IL-33* (**E**), and *IL-13* (**F**) were determined using the 2^−ΔΔCt^ method normalized to *GAPDH*. **G** Representative images of IHC for IL-13 (brown indicated by black arrowheads in the lower panel; scale bars 100 μm for the upper panel and 50 μm for the lower panel); percentage of IL-13-positive area was semi-quantified using Image J. Data are presented as mean + SD for the right panel of **A**, **B**, **G**) and (**C**–**F**). Group size of *n* = 7 per group. ^*^p < 0.05; ^**^p < 0.01; ^***^p < 0.001

Tuft cells have become important sentinels in the intestine, guiding the host’s response to specific damage, including defense against parasitic worm infections and the repair of intestinal epithelium after injury. We examined the marker Dclk1 of tuft cells and found that the number of tuft cells and the transcription level of secretion factor *IL-25* were both increased in the ESPs group. ESPs intervention also inhibited the reduction in tuft cells and the transcription level of *IL-25* caused by DSS (Fig. 7A–D). When the intestinal epithelium is damaged, it secretes the IL-33 to alert the host. We found that both the ESPs and DSS groups exhibited elevated transcription levels of *IL-33*, while the DSS + ESPs group showed a reduction in the transcription level of *IL-33* (Fig. 7E). IL-25 and IL-33 both act on type II innate lymphoid cells (ILC2), promoting them to secrete type 2 cytokines, with IL-13 playing a role in the proliferation and differentiation of ISC. We then assessed IL-13 expression using RT-qPCR and IHC. The results showed that IL-13 secretion was increased in the ESPs group, while it was decreased in the DSS group. ESPs intervention inhibited the DSS-induced reduction in IL-13 (Fig. 7F–G). These findings suggest that ESPs can promote the increase of tuft cells and IL-13 in the host’s small intestine, thereby alleviating UC in mice.

### ESPs promote the proliferation of ISC in the small intestine of UC mice via the tuft/IL-13 signaling pathway

**Fig. 8:**
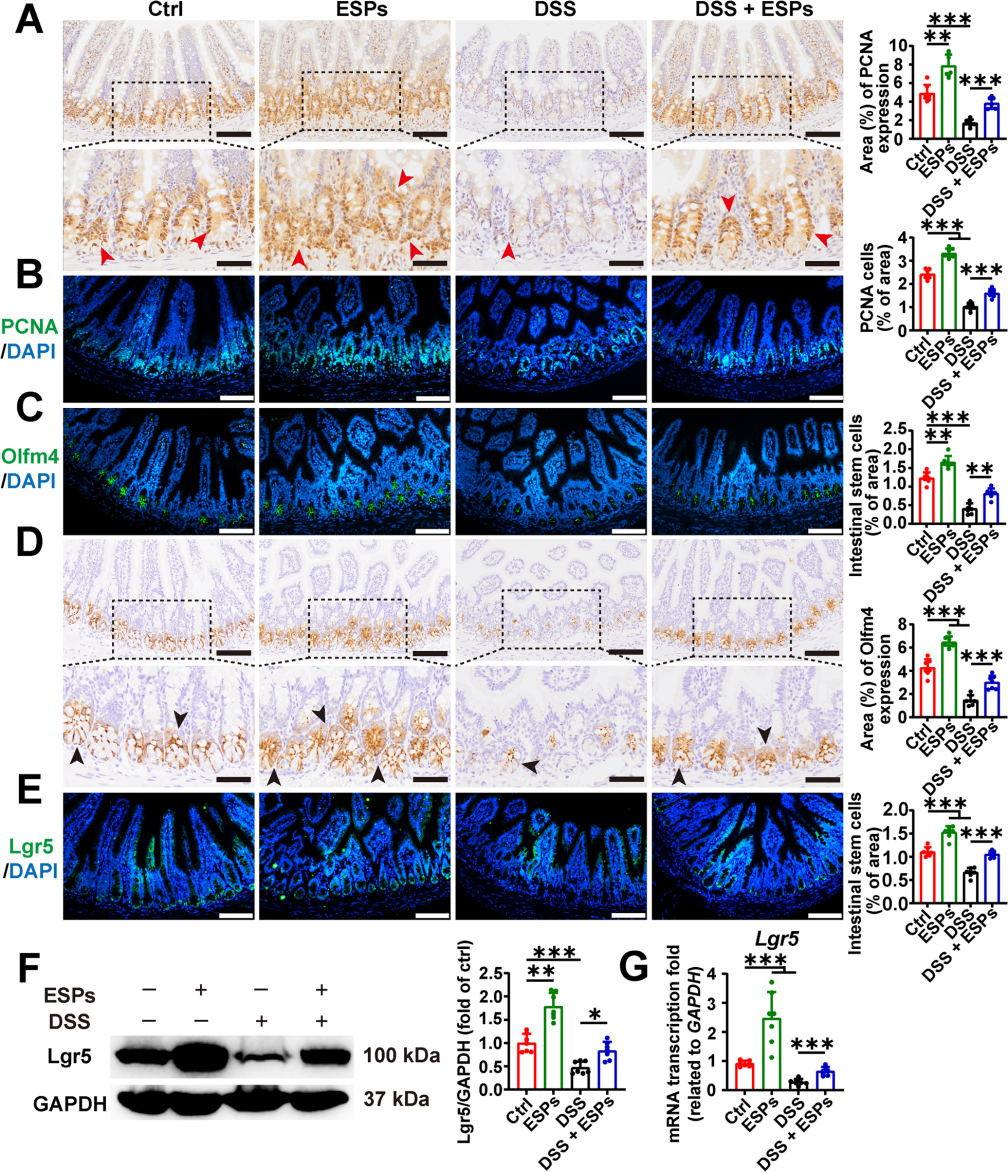
ESPs promote ISC proliferation in the mouse small intestine to alleviate UC. Representative images of IHC for PCNA (**A**) and Olfm4 (**D**); percentages of PCNA and Olfm4-positive areas were semi-quantified using Image J in **A** and **D** (brown indicated by red arrowheads showing PCNA and black arrowheads showing Olfm4 in the lower panels; scale bars 100 μm for the upper panels and 50 μm for the lower panels). Representative images of IF for PCNA (**B**) (green), Olfm4 (**C**) (green), and Lgr5 (**E**) (green) and the nucleus (4′,6-diamidino-2-phenylindole (DAPI), blue) (scale bars 100 μm); percentages of the number of PCNA cells, Olfm4 ISC, and Lgr5 ISC were semi-quantified using Image J in **B**, **C**, and **E**. (**F**) WB result for Lgr5 (left panel) and the relative expression level of Lgr5 (right panel), which was semi-quantified using Image J. (**G**) The transcription level of *Lgr5* was determined using the 2^−ΔΔCt^ method normalized to *GAPDH*. Data are presented as mean + SD for the right panel of **A**–**F** and **G**. Group size of *n* = 7 per group. ^*^p < 0.05; ^**^p < 0.01; ^***^p < 0.001

As previously observed, ESPs intervention alleviated the DSS-induced decrease in IL-13 secretion, which could act on ISCs to promote their proliferation and differentiation. Therefore, we assessed the proliferating cell marker PCNA and ISC markers (Olfm4 and Lgr5) using IHC, IF, and WB. The results showed that PCNA-positive cells and ISCs were increased in the ESPs group. In contrast, the DSS group exhibited a reduction in both PCNA-positive cells and ISCs, and ESPs intervention restored the numbers of these cells (Fig. 8). These findings suggest that ESPs may promote the proliferation of ISC in the ileum of mice through the tuft/IL-13 signaling pathway, thereby alleviating intestinal damage.

### ESPs increase the number of small intestinal Paneth cells and lysozyme levels to alleviate UC in mice

**Fig. 9:**
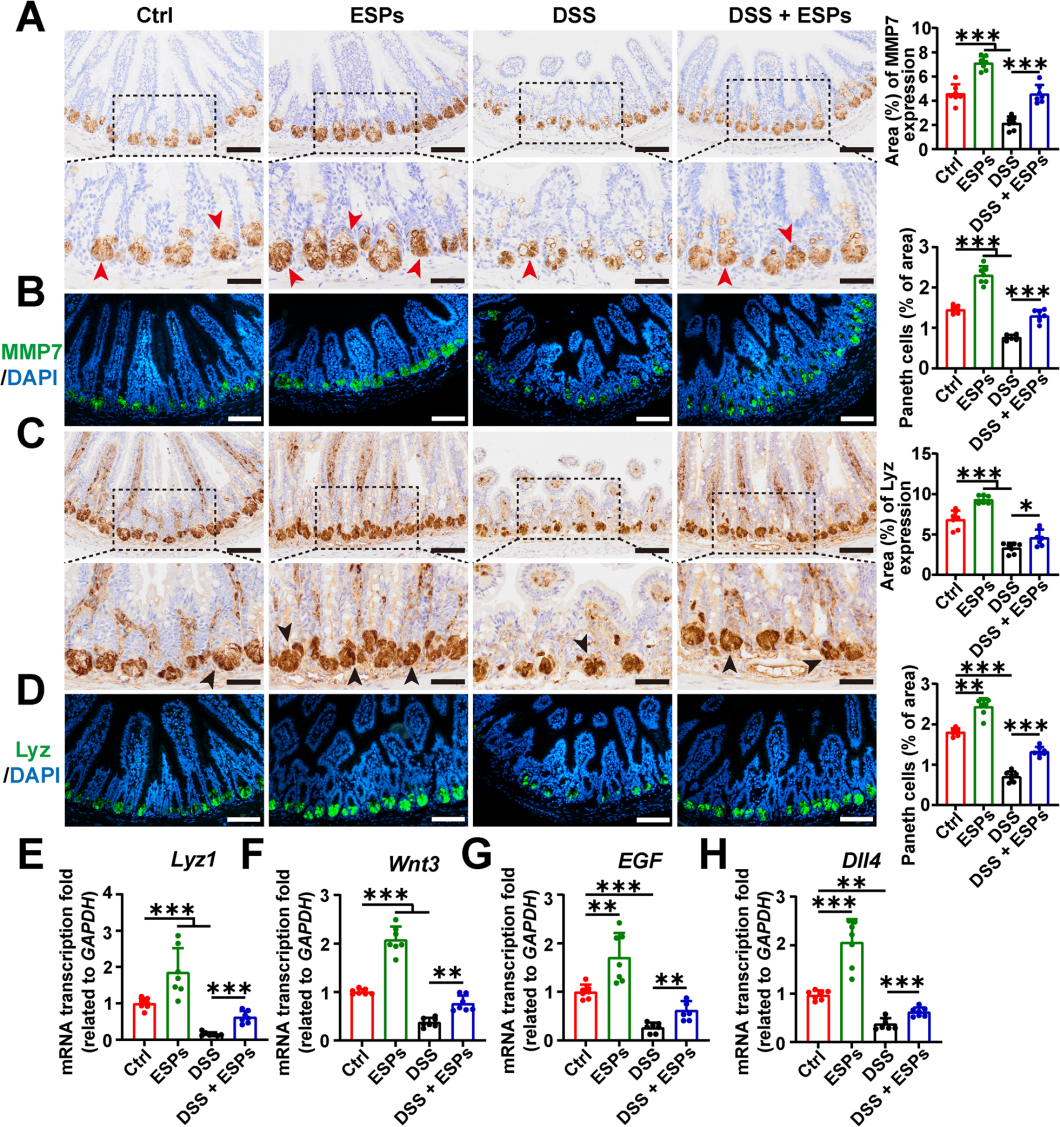
ESPs promote the number of Paneth cells in the mouse small intestine, which secretes lysozyme (Lyz) and growth factors to alleviate UC in mice. Representative images of IHC for MMP7 (**A**) and Lyz (**C**) (brown indicated by red arrowheads showing MMP7 and black arrowheads showing Lyz in the lower panels; scale bars 100 μm for the upper panels and 50 μm for the lower panels); percentages of MMP7 and Lyz-positive areas were semi-quantified using Image J in **A** and **C**. Representative images of IF for MMP7 (green) (**B**) and Lyz (green) (**D**) and the nucleus (4′,6-diamidino-2-phenylindole (DAPI), blue) (scale bars 100 μm); percentage of the number of Paneth cells were semi-quantified using Image J in (**B**) and (**D**). The transcription levels of *Lyz1* (**E**), *Wnt3* (**F**), *EGF* (**G**), and *Dll4* (**H**) were determined using the 2^−ΔΔCt^ method normalized to *GAPDH*. Data are presented as mean + SD for the right panel of **A**–**D** and **E**–**H**. Group size of *n* = 7 per group. ^*^p < 0.05; ^**^p < 0.01; ^***^p < 0.001

We assessed Paneth cells using the marker MMP7 by IHC and IF, and the results showed that ESPs promoted an increase in the number of Paneth cells, whereas DSS damaged the intestinal Paneth cells, leading to a reduction in their numbers. We further used IHC, IF, and RT-qPCR to evaluate another marker (lysozyme (Lyz)) of Paneth cells, and observed that the changes in lysozyme levels were consistent with the findings for Paneth cells (Fig. 9A–E). Paneth cells secrete growth factors to maintain ISC homeostasis, we measured the transcription levels of growth factors *Wnt3*, *EGF*, and *Dll4* via RT-qPCR. Compared with the Ctrl group, the ESPs group showed elevated expression of growth factors, while the DSS group exhibited decreased expression, which was consistent with the changes in Paneth cell numbers (Fig. 9F–H). These results suggest that ESPs can increase the number of Paneth cells and the level of lysozyme in the mouse ileum to maintain intestinal homeostasis, thereby alleviating UC.

### Establishment of an ex vivo culture and co-cultured system for the mouse small-intestine-like organoids

**Fig. 10:**
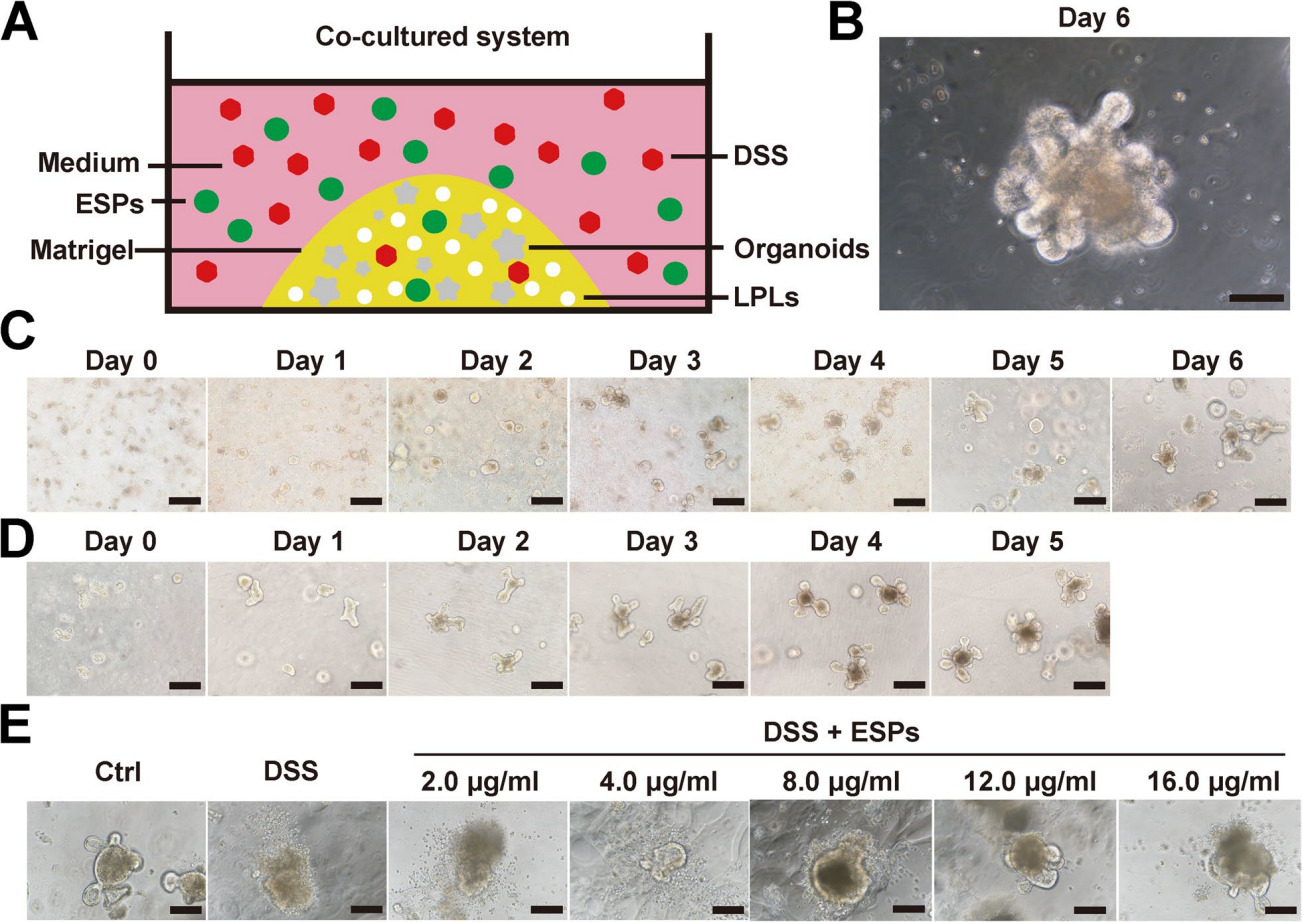
Establishment of a co-cultured model consisting of intestinal organoids and LPLs. (**A**) Schematic diagram of the mouse small-intestine-like organoids co-cultured system. (**B**) Cultured mouse small-intestine-like organoids were observed under the microscope at the sixth day (scale bar 100 μm). (**C**) The growth status of primary organoids were observed by optical microscope at 0–6 days (scale bars 200 μm). (**D**) The growth status of post-passage organoids were observed by optical microscope at 0–5 days (scale bars 200 μm). (**E**) Microscopic observation of the protective effect of different doses of ESPs on DSS-induced small intestinal organoid injury in mice (scale bars 100 μm)

To study the effects of ESPs in vitro, we first co-cultured mouse small intestine organoids with small intestinal LPLs, and induced inflammation in the organoids with DSS, followed by co-culturing with ESPs (Fig. 10A). We observed the morphology of the organoids daily under a microscope. The mouse crypts gradually developed into the complex crypt–villus structure. Successful organoid cultures exhibited distinct budding structures resembling “petals” (Fig. 10B, C), and the organoids showed faster growth after passaging (Fig. 10D). DSS induced morphological damage to the organoids, preventing them from maintaining a complete structure. However, ESPs intervention at different doses alleviated DSS-induced damage to the organoids, with the 12 μg/mL ESPs dose being the most effective. Therefore, this dose was selected for subsequent experiments (Fig. 10E).

### ESPs attenuate organoid damage by increasing tight junction proteins and reducing inflammatory responses

**Fig. 11:**
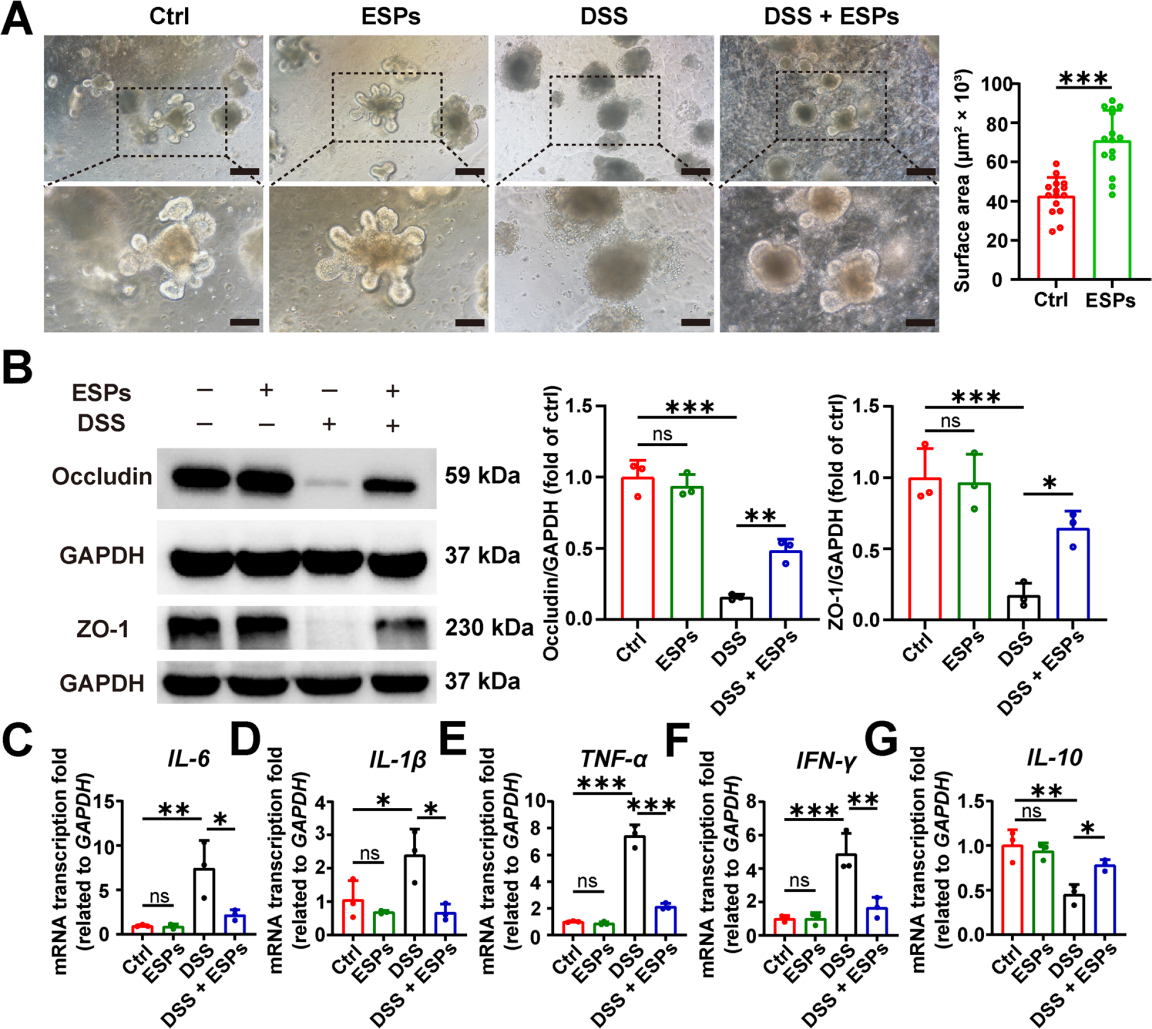
ESPs alleviate organoid damage by maintaining intestinal mucosal barrier integrity and suppressing inflammatory responses. (**A**) Microscopic observation of the protective effect of ESPs on DSS-induced small intestinal organoid injury in mice (scale bars 200 μm for the upper panel and 100 μm for the lower panel) (left panel); percentage of the organoid surface area size (µm^2^ × 10^3^) in the Ctrl and ESP groups were measured using Image J (right panel). (**B**) WB results for occludin and ZO-1 (left panel) and the relative expression of occludin and ZO-1 were semi-quantified using Image J (right panel). The transcription levels of *IL-6* (**C**), *IL-1β* (**D**), *TNF-α* (**E**), *IFN-γ* (**F**), and *IL-10* (**G**) were determined using the 2^−ΔΔCt^ method normalized to *GAPDH*. Data are presented as mean + SD for the right panel of **A**–**B** and **C**–**G**. Group size of *n* = 15 for the right panel of **A**, and *n* = 3 for the right panels of **B** and **C**–**G**. ^*^p < 0.05; ^**^p < 0.01; ^***^p < 0.001; ns, not statistically significant

ESPs could promote the germination of small intestinal organoids in mice, and the disruption of organoid morphology induced by DSS could be alleviated to a certain extent by ESPs (Fig. 11A). The reduction of tight junction proteins (occludin and ZO-1) in small intestinal organoids induced by DSS could be inhibited by ESPs, while ESPs had no significant effect on tight junction proteins in small intestinal organoids (Fig. 11B). In addition, ESPs reduced the transcription levels of pro-inflammatory factors (*IL-6*, *IL-1β*, *TNF-α*, and *IFN-γ*) and increased the transcription level of anti-inflammatory factor *IL-10* to alleviate DSS-induced organoid damage (Fig. 11C–G). The above results suggest that ESPs can maintain the integrity of the intestinal mucosal barrier by increasing the expression of tight junction proteins while attenuating the inflammatory responses, thereby alleviating organoid damage.

### ESPs promote ISC proliferation and differentiation to alleviate DSS-induced organoid damage

**Fig. 12:**
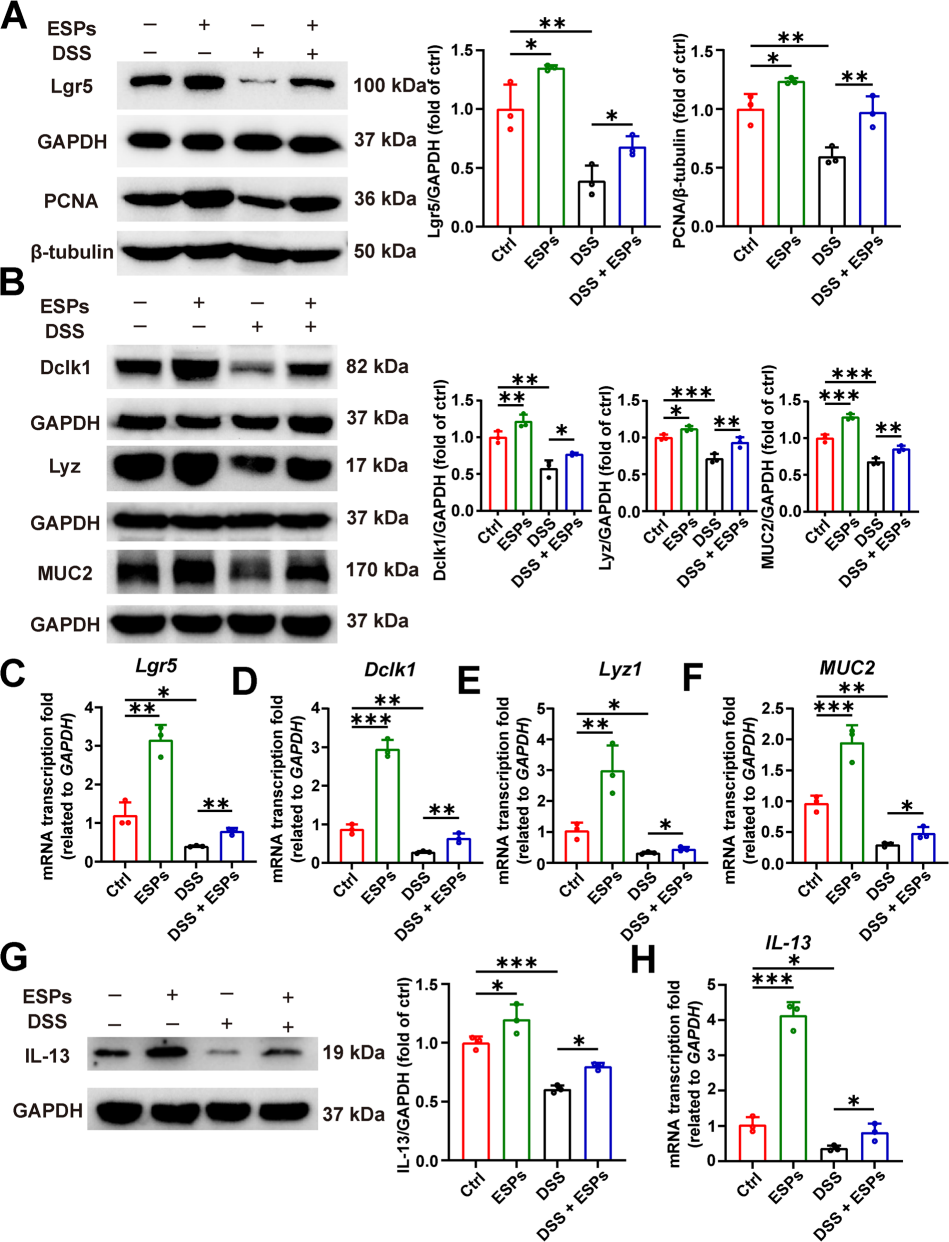
ESPs enhance ISC proliferation and differentiation to promote intestinal epithelial regeneration and alleviate organoid damage. (**A**) WB results for Lgr5 and PCNA (left panel) and the relative expression levels of Lgr5 and PCNA (right panel) were semi-quantified using Image J. (**B**) WB results for Dclk1, Lyz, and MUC2 (left panel); the relative expression levels of Dclk1, Lyz, and MUC2 were semi-quantified using Image J. The transcription levels of *Lgr5* (**C**), *Dclk1* (**D**), *Lyz1* (**E**), *MUC2* (**F**), and *IL-13* (**H**) were determined using the 2^−ΔΔCt^ method normalized to *GAPDH*. (**G**) WB result for IL-13 (left panel) and the relative expression level of IL-13 was semi-quantified using Image J. Data are presented as mean + SD for the right panels of **A**, **B** & **G** and **C**–**F** and **H**. Group size of *n* = 3 per group. ^*^p < 0.05; ^**^p < 0.01; ^***^p < 0.001

The expression of ISC marker Lgr5 and proliferating cell marker PCNA were both increased in the ESPs group compared with the Ctrl group, whereas the expression was decreased in the DSS group, the expressions of Lgr5 and PCNA were restored to a certain extent in the DSS + ESPs group (Fig. 12A), and the results of RT-qPCR also verified that the intervention of ESPs could inhibit the reduction of ISC induced by DSS (Fig. 12C). We detected the tuft cell marker Dclk1, Paneth cell marker Lyz, goblet cell marker MUC2, and cytokine IL-13 by WB and RT-qPCR. DSS caused a decrease in the markers of tuft cell (Dclk1), Paneth cell (Lyz), goblet cell (MUC2), and IL-13, and the intervention of ESPs increased the expression of the Dclk1, Lyz, MUC2, and IL-13 (Fig. 12B–H). The above results suggest that ESPs can promote the proliferation and differentiation of ISC, thereby protecting the intestinal epithelium to alleviate DSS-induced damage to intestinal organoids.

## Discussion

UC is an incurable and recurrent inflammatory bowel disease characterized by chronic diarrhea, abdominal pain, and weight loss [29]. The prevalence of UC is continuously rising, but current conventional medications lack efficacy [30, 31]. The search for methods with fewer side effects and greater effectiveness has become an urgent issue. Previous studies have described the protective effects of worm infections, ESPs, or worm-derived proteins in animal models and placebo-controlled human clinical trials for UC [17, 32, 33]. This suggests that helminths and their related products have potential applications in the treatment of UC. In this study, we explored the alleviating effects of ESPs on UC through the DSS-induced acute UC mouse model and the mouse small intestinal organoid and co-cultured LPL model. The results showed that ESPs significantly reduced the clinical symptoms and pathological damage of DSS-induced UC in mice and alleviated damage in small intestinal organoids. The mechanism might be to induce the proliferation and differentiation of ISC by activating the tuft/IL-13 signaling pathway and to protect the integrity of the intestinal mucosal barrier by increasing the expression of mucins and tight junction proteins, thereby alleviating UC in mice.

The pathogenesis of UC remains unclear, but a major pathological foundation for UC is the impaired intestinal mucosal barrier function and the disruption of tight junction protein integrity. Mucosal healing is associated with improved UC treatment outcomes and has become a key therapeutic target [34]. The intestinal mucosa is primarily composed of tight junction proteins, mucus, antimicrobial peptides, and immune cells or molecules [35]. Tight junction proteins (ZO-1, occludin, and claudin) are major factors influencing changes in the intestinal epithelial barrier in UC [36]; the increase in tight junction protein levels helps maintain the structural integrity of the intestinal barrier, thereby preventing colitis [37]. Mucin MUC2 secreted by goblet cells is a major component of intestinal mucus [38]. One potential cause of UC disease exacerbation is the reduced number of goblet cells in the intestinal mucosa and impaired mucus secretion [39]. Our results showed that DSS induced pathological damage to the colon and ileum in mice. ESPs intervention had no effect on the colon, but it caused shortening of the ileal intestinal villi. Meanwhile, ESPs intervention attenuated DSS-induced clinical signs and pathological impairments in mice. The host could defend against external damage by secreting mucus, but due to excessive goblet cell depletion in the colon induced by DSS, mucus secretion in the colon was reduced. In addition, ESPs could protect the integrity of the intestinal mucosal barrier by inhibiting the abnormal reduction of tight junction proteins caused by DSS. In summary, ESPs had a mitigating effect on UC in mice by protecting the intestinal mucosal barrier integrity.

The interaction between mucosal immunity and intestinal epithelial cells is crucial for maintaining intestinal homeostasis and the innate immune defense barrier of the gut. ISCs can differentiate into various mature intestinal epithelial cells and play a key role in the rapid regeneration of the intestinal epithelium [40, 41]. We examined the changes in ISC and proliferating cells and found that ESPs intervention significantly increased the number of ISCs in the colon and ileum of mice. By assessing the number of proliferating cells with PCNA, we observed that ESPs increased the number of PCNA-positive cells in UC mice, suggesting that ESPs protected the intestinal mucosal barrier by regulating ISC proliferation. Tuft cells in the intestine are relatively few in number, but they play an important role in defense against intestinal worms and intestinal damage. Tuft cells secrete IL-25, which promotes ILC2 secreting IL-13, thereby stimulating the proliferation and differentiation of ISC [42, 43]. In the ileum, DSS induced a reduction in tuft cells and IL-13 secretion, but ESPs intervention increased the number of tuft cells, which in turn enhanced IL-13 secretion. This process might promote ISC proliferation and differentiation. These results suggested that ESPs alleviated DSS-induced colitis by promoting the proliferation of ISCs.

Intestinal Paneth cells secrete antimicrobial peptides to maintain gut homeostasis [44] and regulate ISCs function through the secretion of Wnt3, EGF, and Notch ligands such as Dll4 [45]. In UC, Paneth cells in the ileum undergo metaplasia when they appear in the colon. The lysozyme secreted by metaplastic Paneth cells exacerbates UC, and thus reducing the levels of lysozyme in the colon helps alleviate UC [42]. Moreover, inflammatory factors play a crucial role in the pathogenesis of UC and are closely linked to the severity of inflammation [46]. In the study, we found elevated lysozyme levels and inflammatory responses in the colon of UC mice. ESPs intervention reduced the expression of lysozyme and pro-inflammatory factors while increasing the expression of anti-inflammatory factors. The IL-10 in the DSS + ESPs group was higher than that in the Ctrl group, which might be due to the host inflammation being so severe that pro-inflammatory factors inhibited the secretion of IL-10, which led to a decrease in secretion [47]. IL-10 plays an important function in inflammatory bowel disease by limiting excessive inflammatory responses and promoting tissue repair [48]. When the host’s intestinal pathology was somewhat recovered, the host might try to maintain immune tolerance and prevent relapse by increasing IL-10, which caused an increase in IL-10 secretion, some studies have also showed higher levels of IL-10 in treated UC than in the control group [49, 50]. Restoration of small intestinal Paneth cell function helps alleviate intestinal damage [51]. DSS-induced damage led to a decrease in Paneth cell numbers and lysozyme levels in the ileum, thereby disrupting intestinal homeostasis. ESPs was able to mitigate these pathological changes induced by DSS, promoting Paneth cells secreting lysozyme and growth factors to help restore intestinal homeostasis. The above results showed that ESPs had the potential to ameliorate UC in mice by enhancing lysozyme secretion in the small intestine while inhibiting it in the colon.

The development of three-dimensional (3D) organoid technology has opened a new era for constructing disease models in vitro, which may better represent in vivo conditions [52]. Intestinal organoids provide an excellent model for investigating the mechanisms related to ISC and epithelial repair. Organoids can simulate organ development and demonstrate the self-renewal properties of ISC, thus increasing the authenticity and reliability of the study [53]. The results showed that ESPs could promote the growth of small intestine organoids damaged by DSS-induced inflammation. ESPs treatment increased the expression of tight junction proteins, the ISC marker Lgr5, proliferation cell marker PCNA, tuft cell maker Dclk1, Paneth cell maker Lyz, and goblet cell maker MUC2, while also reducing the inflammatory responses. The in vitro organoid experiment results suggested that ESPs could stimulate the proliferation and differentiation of ISC to repair the damaged intestinal epithelium.

In conclusion, we found that ESPs intervention promoted the proliferation and differentiation of ISCs into a variety of intestinal epithelial cells through the tuft/IL-13 signaling pathway, and these cells secrete mucins, cytokines, and lysozymes to maintain intestinal homeostasis, thereby alleviating UC in mice. These results suggested that ESPs intervention might be beneficial for mucosal healing in UC.

## Conclusions

**Fig. 13:**
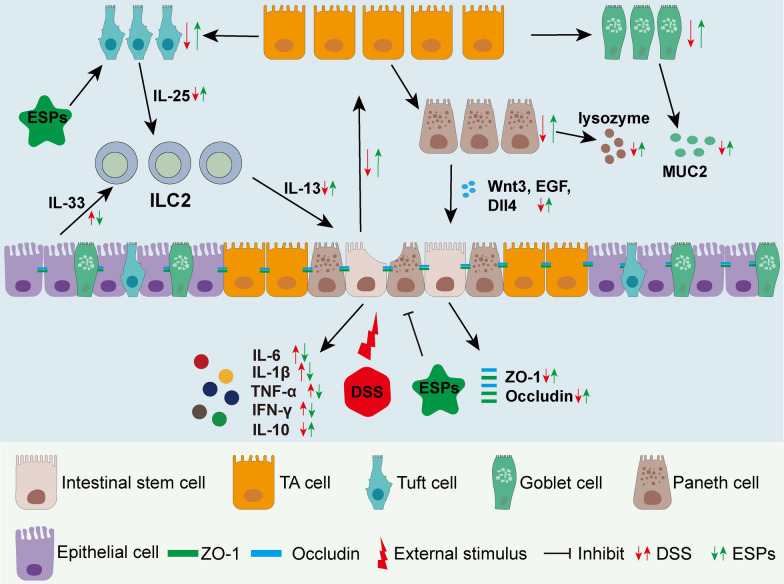
Pathways of action of ESPs intervention to alleviate UC in mice. DSS causes damage to the intestinal epithelium and affects the proliferation and differentiation of ISC in mice, and ESPs intervention promotes regeneration of ISC and intestinal epithelial cells via tuft/IL-13 signaling pathway and attenuates UC in mice

In this study, we found that ESPs can activate the tuft/IL-13 signaling pathway to promote ISC regeneration and protect the integrity of the intestinal mucosal barrier, thereby alleviating UC disease in mice (Fig. 13). This research preliminarily revealed the alleviating effect of ESPs on UC in mice, laying the foundation for future innovative therapies that use worms and their associated products to maintain intestinal mucosal integrity as a treatment for UC.

## Supplementary Information

Additional file 1: Supplementary reagents and methods.
Additional file 2: Figure S1. Identification of *H*. *nana* and ESPs.
Additional file 3: Figure S2. Mitigating effects of different doses of ESPs on UC in mice.
Additional file 4: Table S1. Scoring system for DAI in the mice. Additional file 5: Table S2. Primer sequences used for RT-qPCR.

## Data Availability

Data are provided within the manuscript or supplementary information files.

## Abbreviations

ESPs: Excretory/secretory products
DSS: Dextran sulfate sodium
DAI: Disease activity index
MPO: Myeloperoxidase
ISC: Intestinal stem cell
EGF: Epidermal growth factor
H&E: Hematoxylin–eosin
AB-PAS: Alcian blue–periodic acid–Schiff
RT-qPCR: Real-time quantitative polymerase chain reaction
IHC: Immunohistochemistry
IF: Immunofluorescence
WB: Western blotting
