# Supplementary material for: Excretory/secretory products from Hymenolepis nana adult worms alleviate ulcerative colitis in mice via tuft/IL-13 signaling pathway
Source: Parasit Vectors. 2025 Jun 20;18:230. doi: 10.1186/s13071-025-06893-x (PMC12180163; doi:10.1186/s13071-025-06893-x)
Supplement: Supplementary file 1 — Additional file 1: Supplementary reagents and methods. Additional file 2: Figure S1. Identification of H. nana and ESPs. Additional file 3: Figure S2. Mitigating effects of different doses of ESPs on UC in mice. Additional file 4: Table S1. Scoring system for DAI in the mice. Additional file 5: Table S2. Primer sequences used for RT-qPCR. [file 13071_2025_6893_MOESM1_ESM.docx]

**Supplementary materials for**

Excretory/secretory products from *Hymenolepis* *nana* adult worms alleviate ulcerative colitis in mice via tuft/IL-13 signaling pathway

Rong Mou, Xuanyin Cui, Hongyan Wang, Zhenfen Zhang, Yi Cheng, Wenlan Wu, Jinfu Li, Ke Zhang

*Email: kevin312_112@aliyun.com (K. Z.)

**Reagents and methods**

**1. Reagents**

DAB staining kit (ZLI-9018, zsbio), dextran sulfate sodium (DSS, MW: 36 kDa-50 kDa, MP), carmine (1390-65-4, Macklin), Myeloperoxidase assay kit (A044-1-1, Nanjing Jiancheng) were offered by Guiyang Jingong Technology Co (Guiyang, China). Masson's Trichrome Stain Kit (G1343), Hematoxylin-Eosin Stain Kit (G1120), EDTA antigen retrieval solution (C1034), Fecal Occult Blood Kit (BC8270), 1M HEPES (H1095), Percoll cell separation solution (P8370), 1M DTT solution (D1070), and DAPI solution (C0065) were offered by Solarbio (Beijing, China). AB-PAS Stain Kit (R22021) was obtained from Saint-Bio (Shanghai, China). RT-qPCR Reverse Transcription Kit (11141ES60), SYBR RT-qPCR Kit (11201ES08), RIPA lysis buffer (20101ES60), BCA Protein Quantification Kit (20201ES76), TRIeasy Total RNA Extraction Reagent (10606ES60), MolPure Cell/Tissue DNA Kit (18700ES50), collagenase IV (40510ES60), and Super enhanced chemiluminescence (ECL) Detection Reagent (36208ES60) were provided by Yeasen (Shanghai, China). Goat serum (abs933) was purchased from Absin (Shanghai, China). DNase I (D7073) was offered by Beyotime (Shanghai, China). Mouse Intestinal Organoid Kit (K2001-MI) and Organoid Culture ECM (Matrigel) (Reduced Growth Factor) (M315066) were offered by bioGenous (Jiangsu, China).

**2. The extraction of excretory/secretory products (ESPs) from *H*. *nana* adult worms**

The *H*. *nana* obtained from the hamster's small intestine was rinsed several times with sterilized PBS. Subsequently, 20-30 adult worms were immersed in RPMI 1640 medium, supplemented with 1% Penicillin/Streptomycin/Amphotericin B. This suspension was incubated at 37°C for 2 h within a thermostat-controlled incubator. Following this initial incubation, the medium was refreshed and the worms were further incubated for 36 h. The conditioned medium containing ESPs was then collected. To concentrate the ESPs-rich medium, centrifugation at 4,000 x g was executed utilizing a 10 kDa ultrafiltration tube (UFC901096, Millipore, Billerica, MA, USA), with the solvent subsequently exchanged for PBS. Sterility was ensured by passing the ESPs solution through a 0.22 μm filter, and the protein content was quantified using a BCA protein assay. The prepared ESPs was then stored at -80°C for future applications.

**3. Identification of *H. nana* and ESPs, and the acquisition of serum from *H*. *nana-*infected hamster**

A few parasites were randomly selected to obtain eggs by cutting up the nodes, and the adults were stained with carbolic acid red (configured with carmine), eggs and stained adults were observed using an upright fluorescence microscope (Eclipse 80i, Nikon Ltd, Japan). A few parasites were re-selected and worm DNA was extracted using a MolPure Cell/Tissue DNA Kit, and PCR amplification of the *COX-I* gene of *H*. *nana* followed by agarose gel electrophoresis.

For serum from *H*. *nana* infected hamsters, blood samples were obtained from the eyeballs of *H*. *nana* infected hamsters, stored at room temperature for 2 h, centrifuged at 10,000 x g for 10 min, then aspirated the serum and stored at -80°C. Meanwhile, the number and molecular size of proteins contained in ESPs were evaluated by immunoblotting. Briefly, 5% stacking gel and 10% resolving gel were meticulously prepared. Each well of the gel was loaded with 15 µg of protein. After electrophoresis, the protein samples were transferred to a PVDF membrane, which was subsequently blocked with 5% skim milk powder to minimize nonspecific binding. Primary antibody (serum from *H*. *nana* infected hamster, diluted to 1:50) was applied to the membrane and incubated overnight. At the next day, an anti-mouse secondary antibody (1:10,000) was added and incubated for 1 h. Finally, Super ECL Detection Reagent was utilized for sensitive detection of the protein.

Initially, we identified the parasite through morphological and molecular biology techniques, observing that the eggs were round or nearly round, containing a hexacanth embryo; the adult's scolex had suckers and rostellum; the proglottids contained ovaries and testes; the gravid proglottids were filled with eggs. PCR amplification of the *COX-I* gene of *H*. *nana* showed a clear band at 202 bp, confirming that the parasite used in this study was indeed *H*. *nana* (Additional file 2: Figure S1B-F). The results revealed that there were many proteins in the ESPs, but four major bands with relatively high expression levels were observed, located at 20-25 kDa, 35-45 kDa, 45-60 kDa, and 100-140 kDa (Additional file 2: Figure S1G).

**4.** **Fecal occult blood test**

Pick fresh mouse feces on a white porcelain plate, according to the Fecal Occult Blood Kit operation, add drops of the corresponding reagent on the feces, immediately timing and observing the colour change, and finish the reading within 2 min. 0 point for no colour development within 2 min; 1 point for gradual change of light green to green after 10 s; 2 points for light green at first after the addition of the reagent, and then gradually obvious blue-brown; 3 points for blue-brown immediately after the addition of the reagent, and then gradually black brown; 4 points for blue-black-brown immediately after the addition of the reagent.

**5. Crypt isolation and extraction of intestinal lamina propria lymphocytes (LPLs)**

After anesthetizing and euthanizing 6-8 weeks-old mice, the ileum tissues were extracted, and the intestinal villi were scraped off using a slide. The ileum tissues were then washed several times with sterile PBS until the supernatant turned clear, and the tissues were cut into 2 mm fragments. For crypt extraction, the intestinal tissues were digested on ice for 20 min in 5 mM EDTA, washed three times with DPBS, and gently sucked and blown with a Pasteur pipette. A small amount of suspension was observed under a microscope, and once a substantial number of crypts were visible, the suspension was filtered through a 70 μm cell strainer. For the extraction of LPLs, the intestinal tissues were incubated at 37°C in an EDTA solution (1 x DPBS, 5% FBS, 1% penicillin/streptomycin, 10 mM HEPES, 1% L-glutamine, 10 mM EDTA, 1 mM DTT) for 20 min. The supernatant was discarded, and the tissues were further incubated at 37°C in a digestion solution (RPMI 1640, 1% L-glutamine, 10mM HEPES, 1% penicillin/streptomycin, 5% FBS, 1 mg/mL collagenase IV, and 1 U/mL DNase I) for 30 min. Vortex mixing was applied for thorough agitation, and the supernatant was filtered through a 70 μm cell strainer. The filtrate was centrifuged at 200 x g for 5 min, and the pellet was resuspended in 40% percoll and then layered over 80% percoll. After centrifuging at 500 x g for 20 min, the interface containing LPLs was carefully collected.

**6. Collection of mouse small intestinal organoids**

Discard the culture medium from the 24-well plate, add 1 mL of pre-chilled DPBS to wash once, discard the DPBS, and add another 1 mL of pre-chilled DPBS. Use sterilized scissors to cut off the tips of pipette tips and gently break up the Matrigel. Transfer the intestinal organoids into a 15 mL centrifuge tube using a pipette, pipette up and down for several times, then place the tube at −20 °C for 5 min, followed by centrifugation at 300 x g for 5 min at 4 °C. Discard the supernatant and retain the pellet; repeat the centrifugation once. The final pellet is the intestinal organoids and store at -80°C for subsequent experiments.

**7. Extraction of tissues total RNA and reverse transcription to cDNA**

Extract total RNA from mouse colonic, ileum, and small intestinal organoid tissues with 1 mL TRIeasy Total RNA Extraction Reagent, then add 200 μL trichloromethane, and centrifuge at 4°C 13,000 x g for 20 min, take the supernatant, add an equal amount of isopropanol, mix well and centrifuge at 4°C 13,000 x g for 10 min, discard the supernatant, and the precipitate at the bottom will be total RNA, add 75% ethanol to wash the RNA precipitate, centrifuge at 4°C 5,400 x g for 10 min, and finally add 30-50 μL DEPC water.

The concentration of RNA was detected using a NanoDrop 2000 UV-vis spectrophotometer (Thermo Fisher Scientific, Waltham, MA, USA). Subsequently, the total RNA was reversely transcribed into cDNA, and the amplification was detected using the SYBR RT-qPCR Kit in a real-time fluorescence quantitative PCR instrument (CFX96, Bio-Rad, Hercules, CA, USA). The amplification protocol consisted of an initial denaturation step at 95°C for 5 min, followed by 39 cycles of denaturation at 95°C for 10 s, and annealing at 60°C for 30 s.

**8. Hematoxylin-eosin (****H&E) staining, Alien-blue and Periodic acid-Schiff (AB-PAS) staining, and Masson's trichrome (Masson) staining**

Mouse ileum and colon tissues were fixed with 4% paraformaldehyde for 24 h. The tissues were embedded in paraffin and then cut into 4 μm thickness sections. Sections were treated with xylene followed by a gradient of ethanol. According to the reagent instructions, H&E staining for histopathologic examination of inflammatory conditions of the colon and ileum, AB-PAS staining for detection of goblet cells in the colon and ileum, and Masson staining for collagen fibers in the colon. After sealing the slices with neutral gum, images were observed and captured using a slide scanner (Olympus SLIDEVIEW VS200, Japan).

**9. Immunohistochemistry (IHC) and immunofluorescence (IF)**

The paraffin-embedded sections of mouse colonic and ileum tissues were first deparaffinized using xylene and graded ethanol. Sections were then repaired by boiling through 1 x EDTA antigen retrieval solution. Following this, Endogenous peroxidase activity was blocked with 3% endogenous peroxidase. For IHC, the sections were blocked with 5% goat serum for 30 min before incubation overnight with primary antibodies specific for ZO-1 (1:2,000), Occludin (1:200), MUC2 (1:2,000), Olfm4 (1:200), Dclk1 (1:200), PCNA (1:200), MMP7 (1:200), Lysozyme (Lyz) (1:2,000), and IL-13 (1:200). On the next day, the sections were incubated with the appropriate secondary antibody (1:500), followed by DAB staining. Finally, the sections were mounted with neutral gum and observed using a slide scanner (Olympus SLIDEVIEW VS200, Japan). For IF, the sections were permeabilized with 0.3% Triton X-100 for 30 min and blocked with 5% BSA for 1 h. They were then incubated overnight with primary antibodies respectively specific for Lgr5 (1:100), PCNA (1:200), Olfm4 (1:200), MUC2 (1:500), Dclk1 (1:200), MMP7 (1:400), and Lysozyme (Lyz) (1:250). On the next day, the sections were incubated with the fluorescent secondary antibody 488 (1:200) and mounted in the DAPI solution. Finally, the sections were mounted with an anti-fade mounting medium and observed using an upright fluorescence microscope (Eclipse 80i, Nikon Ltd, Japan).

**
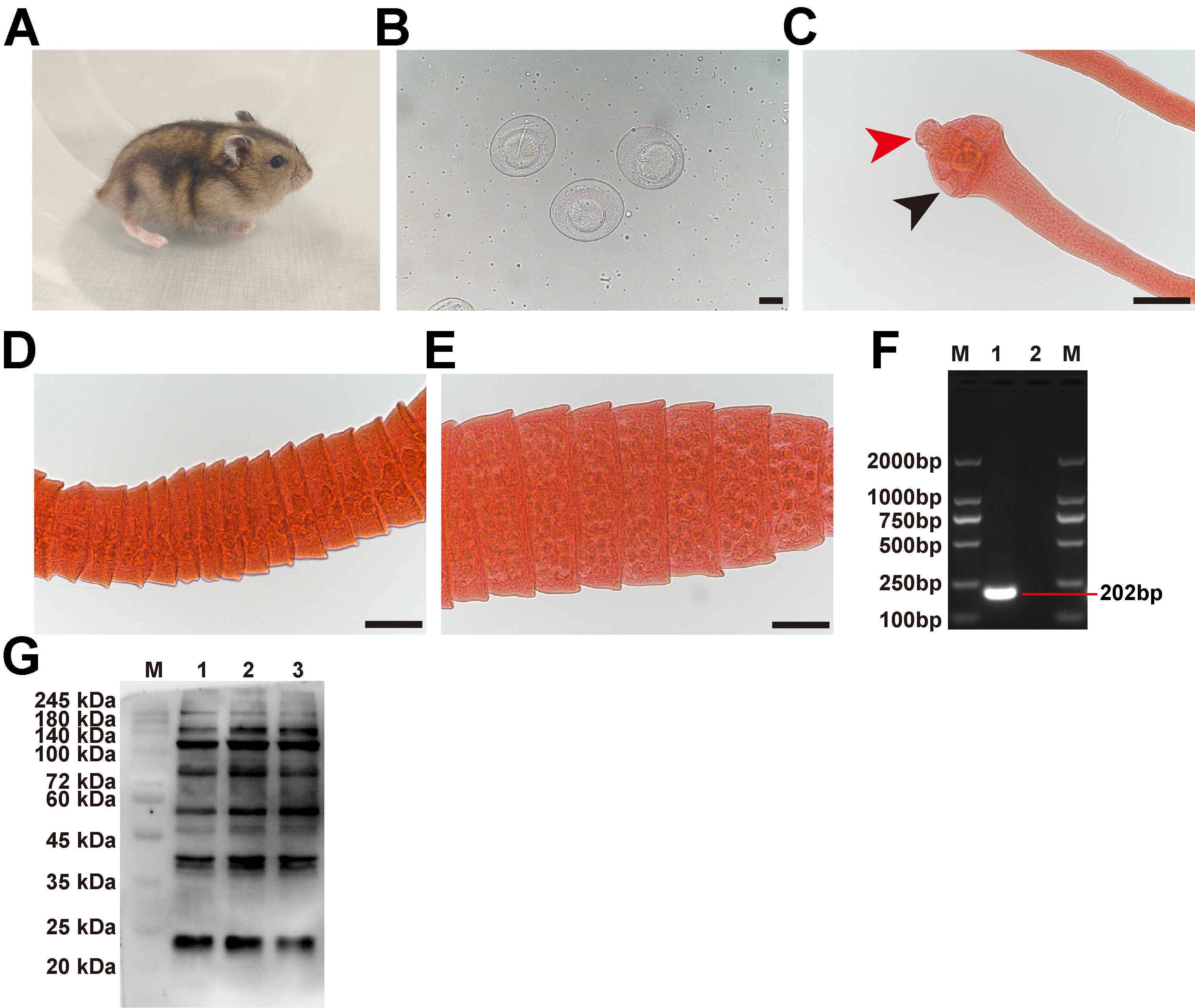
**

**Figure S1. Identification of *H*. *nana* and ESPs.** (**A**) Representative picture of the hamsters from an urban pet market. (**B**) Representative image of the eggs of *H*. *nana* (scale bar 20 μm). (**C**) Representative image of the scolex of *H. nana* (the sucker pointed by a black arrowhead and the rostellum pointed by a red arrowhead, scale bar 100 μm). (**D**) Representative image of mature proglottids of *H*. *nana* (scale bar 100 μm). (**E**) Representative image of gravid proglottids of *H*. *nana* (scale bar 100 μm). (**F**) PCR result of the *COX-I* of *H*. *nana* (F: 5’-ACCGCGTCGTGTGTGTATTT-3’, R: 5’-ACATGCAACTGGGCTCATACG-3’), the proposed amplicon was 202 bp. M: DL 2000 marker, Lane 1: PCR product of the *COX-I* of *H*. *nana*, Lane 2: sterilized H_2_O. (**G**) Immunoblotting result of ESPs. M: protein marker; Lane 1-3: ESPs

**
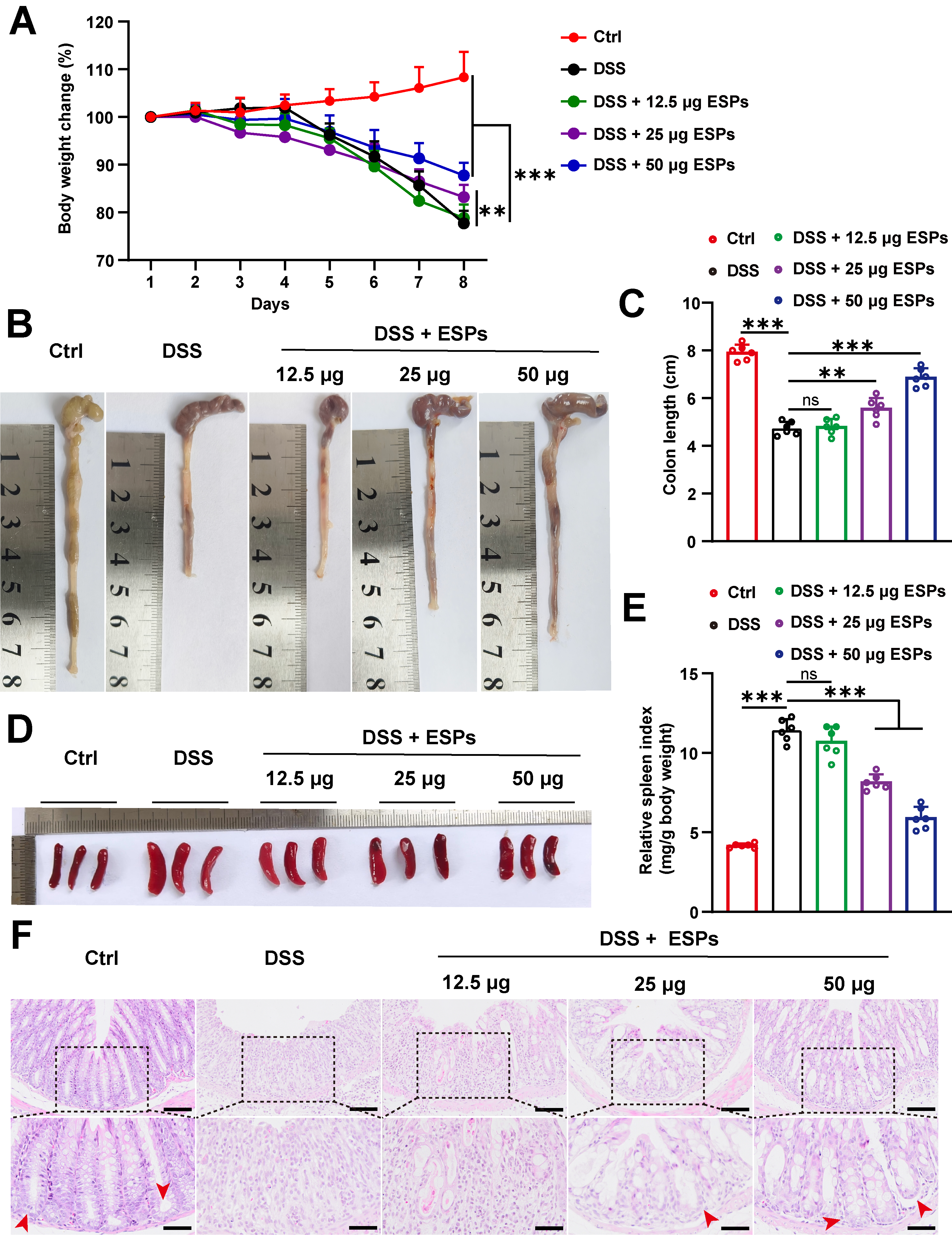
**

**Figure S2. Mitigating effects of different doses of ESPs on UC in mice.** (**A**) Changes in mouse body weight (%). Initial body weight was set as 100%. (**B**) Representative colon images. (**C**) Colon length (cm). (**D**) Representative spleen images. (**E**) Statistics for spleen index. (**F**) Representative images of H&E-stained colon intestine (the crypts pointed by red arrowheads in the lower panel; scale bars 100 μm for the upper panel and 50 μm for the lower panel). Data are presented as mean + SD in (A), (C), and (E), *n* = 6 per group for (A), (C), and (E), ** *p* < 0.01, *** *p* < 0.001, ns: not statistically significant.

**Table S1. Scoring system for DAI in the mice**

| Loss of body weight (%) | Shape of feces | Bloody stools | Score |
| --- | --- | --- | --- |
| <1 | normal | — | 0 |
| 1-5 | loose | （+） | 1 |
| 5-10 | semi-formed loose stool | 2（+） | 2 |
| 10-15 | loose, not attached to the anus | 3（+） | 3 |
| ≥15 | loose, attached to the anus | 4（+） | 4 |

**Table S2. Primer sequences used for RT-qPCR**

| Target Genes | Primer Sequences (5’ - 3’) |
| --- | --- |
| *Lgr5* | F: CCTGGGAAAGCATACCCGTT  R: GGTTGACTCACAGGACCGTT |
| *Lyz1* | F: CCCAAGATCTAAGAATGCCTGT  R: CCCATGCTCGAATGCCTT |
| *Wnt3* | F: GCTGCCAAGAGTGTATTCGC  R: CCGCACAATCTACCCCTTCC |
| *EGF* | F: GTGGCTCCGTCCGTCTTATC  R: GGCTATCCAAATCGCCTTGC |
| *Dll4* | F: AAGGTGCCACTTCGGTTACA  R: GGCAATCACACACTCGTTCC |
| *Dclk1* | F: CAGCCTGGACGAGCTGGTGG  R: TGACCAGTTGGGGTTCACAT |
| *IL-25* | F: CAGCCTGGACGAGCTGGTGG  R: TGACCAGTTGGGGTTCACAT |
| *IL-33* | F: GTATTCCAACTCCAAGATTTCCC  R: CATGCAGTAGACATGGCAGA |
| *IL-6* | F: TGGGACTGATGCTGGTGAC  R: CACAACTCTTTTCTCATTTCCACG |
| *IL-1β* | F: AGCAGCTATGGCAACTGTTC  R: ACAGGTCATTCTCATCACTGTCAA |
| *MUC2* | F: ACCACAATCTCTACTCCCATCT  R: TCCAGTCAGACCAAAAGCAG |
| *TNF-α* | F: ACGGCATGGATCTCAAAG  R: TGGGAGTAGACAAGGTACAACC |
| *IFN-γ* | F: CACACCTGATTACTACCTTCTTCAG  R: GACTCCTTTTCCGCTTCCTGAGG |
| *IL-10* | F: ACCAATAGCTGATGTTGCCA  R: GAATGATGCCAGAGCTACGA |
| *IL-13* | F: AGCTCCCTGGTTCTCTCACT  R: CTCATTAGAAGGGGCCGTGG |
| *GAPDH* | F: AGGAGCGAGACCCCACTAACA  R: AGGGGGGCTAAGCAGTTGGT |
